# TLR25 is endosomally located and responds to *Francisella* infection in Atlantic cod

**DOI:** 10.3389/fimmu.2026.1798290

**Published:** 2026-07-08

**Authors:** Synne Arstad Bjørnestad, Pia Krokene, Monica Hongrø Solbakken, Tannu Priya Gosain, Kjetill S Jakobsen, Sissel Jentoft, Oddmund Bakke, Cinzia Progida

**Affiliations:** 1 Section for Physiology and Cell Biology, Department of Biosciences, University of Oslo, Oslo, Norway; 2 Centre for Ecological and Evolutionary Synthesis (CEES), Department of Biosciences, University of Oslo, Oslo, Norway

**Keywords:** Atlantic cod (Gadus morhua), endolysosomes, Francisella, innate immunity, TLR14, TLR25, TLRs

## Abstract

Atlantic cod possess a unique immune system due to the loss of the MHC II antigen-presenting system and an extreme expansion of the MHC class I repertoire. Additional modifications are demonstrated within the innate immune system with losses and expansions of Toll-like receptor (TLR) genes. While Atlantic cod has lost its mammalian orthologs for plasma membrane localized TLRs, such as TLR1/6 and TLR2, it has expanded its intracellular TLRs including TLR7, -8, and -9. It has been hypothesized that teleost-specific TLR1-family related members TLR14 and TLR25 localize at the plasma membrane with the potential function to recognize bacterial surface components, compensating for the loss of cell surface TLRs. In this study, we have investigated the intracellular localization and function of Atlantic cod TLR14 and TLR25 using super-resolution microscopy in an Atlantic cod cell line and primary cells. Our results show that these TLRs localize to endolysosomes, and colocalize with LysoTracker, Rab5, and Rab7. Interestingly, TLR14 is also present at the plasma membrane, suggesting a function at the cell surface. In cells infected with *Francisella noatunensis* subsp. *noatunensis (Fnn)*, these TLRs colocalize with the internalized bacteria. While the localization of both TLR14 and TLR25 to late endosomal compartments increases upon infection with *Fnn*, only TLR25 relocates to the perinuclear region and triggers up-regulation of the pro-inflammatory cytokines IL-6 and IL-18. Overall, our findings provide novel insight into the function of teleost-specific TLRs in Atlantic cod which indicates that these TLRs employ different strategies for microbial detection and activation.

## Introduction

The innate immune system constitutes the first-line defense against foreign microbial invaders. Central to this system are pattern recognition receptors (PRRs) that recognize and bind pathogen associated molecular patterns (PAMPs) (1). Binding between PRRs and PAMPs initiates downstream signaling pathways, activating the host’s innate as well as affecting the adaptive defence (1, 2). One of the largest PRR families is the Toll-like receptors (TLRs). TLRs are type I transmembrane receptors that recognize bacterial-, fungal-, parasitic-, and viral-specific PAMPs (2). There are 26 vertebrate TLRs of which mammals display TLR1-13, while teleosts additionally display TLR14-28 (3–5). The vertebrate TLR repertoire can be divided into six families: TLR1, -3, -4, -5, -7, and -11. Each family recognizes a general class of PAMPs, with most vertebrates possessing at least one member from each family (6). The TLR repertoire in teleosts is more diverse than in mammals, which reflects their unique evolutionary history and distinct environmental background (7). Even though studies on fish TLRs suggest high structural and functional similarities to mammalian TLRs (7, 8), limited knowledge exists on fish-specific TLRs, with only a few ligands having been determined (4, 7).

Intriguingly, genome analysis of Atlantic cod (*Gadus morhua*) and other teleost species have revealed alternative immune strategies challenging the conventional perspective of a functional immune system (9–12). Around a decade ago, sequencing of the Atlantic cod genome, followed by that of many Gadiformes species, revealed complete loss of the Major histocompatibility complex (MHC) class II and an extreme expansion of the MHC class I system. This was accompanied by equally interesting changes within the TLR gene repertoire characterized by multiple gene losses and expansions (13–16). Expansions of TLR genes correlate with the loss of MHCII (17), and losses relate to cell surface TLRs, including TLR1, -2, and -5, known to recognize bacterial outer components, such as peptidoglycan and lipoproteins, at the plasma membrane. However, Atlantic cod retains TLR14 and TLR25, with expansion of TLR25, that have been proposed to harbor TLR1-family-like function for alleviating the loss of cell surface TLRs (3, 13). In support of this hypothesis, protein structure modelling of TLR14 and TLR25 has revealed a short, disrupted solenoid structure, which is in line with plasma membrane location and non-nucleic acid ligand binding (3, 7, 18). Moreover, the TLR signaling pathways for TLR1-family members are intact in Atlantic cod (3), further supporting the presence of TLRs harboring this function.

Multiple nucleic acid-recognition TLRs, including TLR7, -8, -9, and -22, also show remarkable expansions, giving Atlantic cod one of the highest numbers of TLRs found in teleosts (13, 17). The expansion of TLRs in Atlantic cod most likely increases the detectable ligand repertoire through neo- and sub-functionalization suggesting that TLR losses do not result in an overall reduced ability of pathogen detection (3). However, it has been proposed that the Atlantic cod TLR repertoire may rely more on nucleic acid-detecting TLRs for bacterial pathogen recognition (13).

As TLRs function as a link between the innate and adaptive immune system (19), elucidating the function of the TLR repertoire in Atlantic cod will help us understand the overall immune strategy for this species. Even though previous studies show that TLR14 participates in detection of bacterial ligands in Japanese flounder (*Paralichthys olivaceus*) and Asian swamp eel (*Monopterus albus*) *(*20, 21) and TLR25 in detection of parasites in channel catfish (22), the specific ligands for these fish-specific TLRs and their subcellular localization are not fully elucidated. In this work, we have for the first time revealed the intracellular localization of these TLRs in an Atlantic cod cell line and primary cells. We also investigated the intracellular localization of TLR3, as comparative analyses based on sequence homology have shown high degree of sequence conservation and suggested recognition of nucleic acid-ligands and endosomal location, identical to its mammalian counterpart (3, 4). By using super-resolution microscopy, we have demonstrated that TLR14 is present at both cell surface and endosomes, while TLR25 and TLR3 localize exclusively to endosomes. Moreover, we have determined that the facultative intracellular Gram-negative bacterium *Francisella noatunensis* subsp. *noatunensis (Fnn)* is internalized in TLR3-, -14, and -25-positive endosomes. Bwhileoth TLR14 and TLR25 increase localization to late endosomes upon *Fnn* infection. However, only TLR25 relocates to the perinuclear region and up-regulates the expression of the pro-inflammatory cytokines IL-6 and IL-18.

Altogether, our results confirm that TLR3 is present on late endosomes, equal to its mammalian counterpart, and demonstrate that TLR14 but not TLR25 is present at the plasma membrane. However, while TLR14 appears unresponsive to Gram-negative bacteria in Atlantic cod, our results indicate that TLR25 does respond, suggesting that TLR25 is responsible for detecting bacterial ligands from within endosomes.

## Results

### TLR14 but not TLR25 localizes to the plasma membrane in Atlantic cod

Atlantic cod possesses a TLR repertoire characterized by multiple gene expansions and losses (3, 13). A peculiarity is the loss of TLR1-family members TLR1/6 and TLR2, detecting peptidoglycan and lipoproteins at the plasma membrane (1). TLR14 and TLR25 have been proposed to compensate for this loss by taking over the TLR1-family-like function and localize to the plasma membrane (3). However, this has not been experimentally verified. We therefore investigated if Atlantic cod TLR14 and TLR25 are plasma membrane TLRs by using live-cell super-resolution microscopy. While TLR14 is present as a single gene copy in Atlantic cod (ENSGMOG00000003793), TLR25 has an expansion of nine gene copies (3). However, compared to the other TLR gene expansions, TLR25 displays the least amount of sequence variation, and the majority of this diversity is located to the ectodomain (**Supplementary Figures 1A**, **8**). We therefore selected one TLR25 copy (ENSGMOG00000036237) to further characterize its cellular localization.

**Figure 1:**
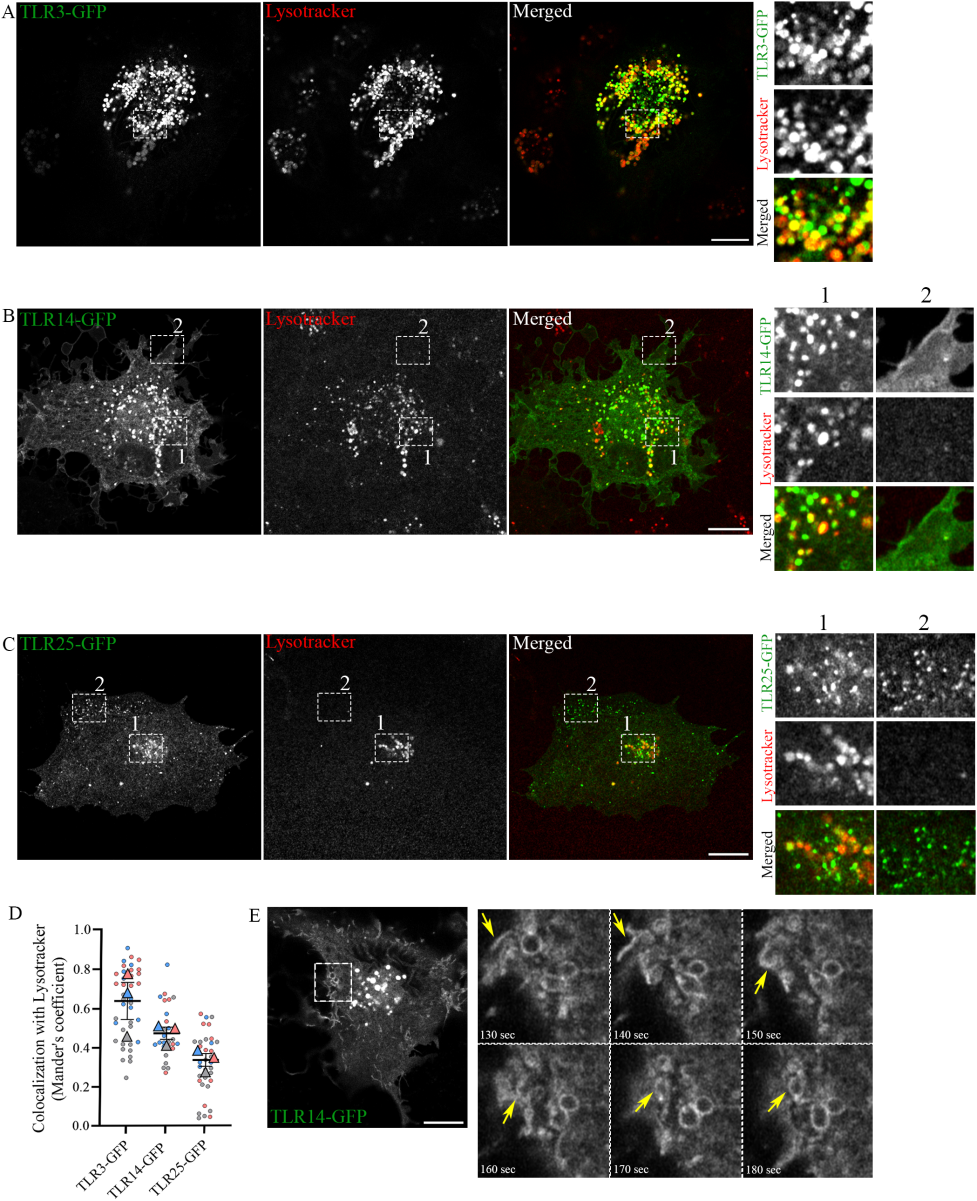
TLR3, TLR14 and TLR25 subcellular localization in ACL cells. Representative images of ACL cells transiently transfected with Atlantic cod **(A)** TLR3-GFP, **(B)** TLR14-GFP, or **(C)** TLR25-GFP, stained with LysoTracker Red and imaged using a Zeiss LSM880 Fast AiryScan microscope. Scale bar: 10 µm. Magnification of boxed areas are shown to the right. **(D)** The graph represents colocalization (Manders’ coefficient) between TLR3, TLR14, or TLR25 and LysoTracker Red. Scatter plot shows the mean ± SEM from three independent experiments. Dots represent individual measurements and are color coded for each experimental repeat. n ≥ 33 cells in total. **(E)** ACL cell transiently transfected with TLR14-GFP and imaged live with a Zeiss LSM880 Fast AiryScan microscope. Arrows in the magnifications of the boxed area point to a membrane ruffle positive for TLR14 that is internalized onto an endosome. Scale bar: 10 µm.

GFP-tagged TLRs were transiently transfected into the Atlantic cod larval (ACL) cell line. Cells were subsequently labelled with LysoTracker Red, a fluorescent dye that accumulates in acidic compartments such as late endosomes and lysosomes. Intriguingly, all TLRs localized to perinuclear vesicles that partially overlapped with acidic, LysoTracker Red-positive, compartments (**Figure 1**). We also included the endosomal, nucleic acid-sensing TLR3 in our analysis, as it is well-conserved across vertebrate species including teleosts (5), and is present as a single gene copy (ENSGMOG00000035330) in Atlantic cod (3). TLR14 was detected at the plasma membrane as predicted, in addition to perinuclear endosomes. In contrast, TLR25 localized exclusively to endosomes (**Figures 1B, C**). Time-lapse video-microscopy of TLR14-GFP clearly demonstrates its location to cell surface ruffles, followed by their closure and internalization into macropinosomes (**Figure 1E**; **Supplementary video 1**). Colocalization analysis shows that TLR3-GFP and TLR14-GFP have the highest overlap with LysoTracker (Mander’s coefficient 0.68 and 0.50, respectively) while TLR25-GFP overlapped the least (Mander’s coefficient 0.37, **Figure 1D**).

**Figure 2:**
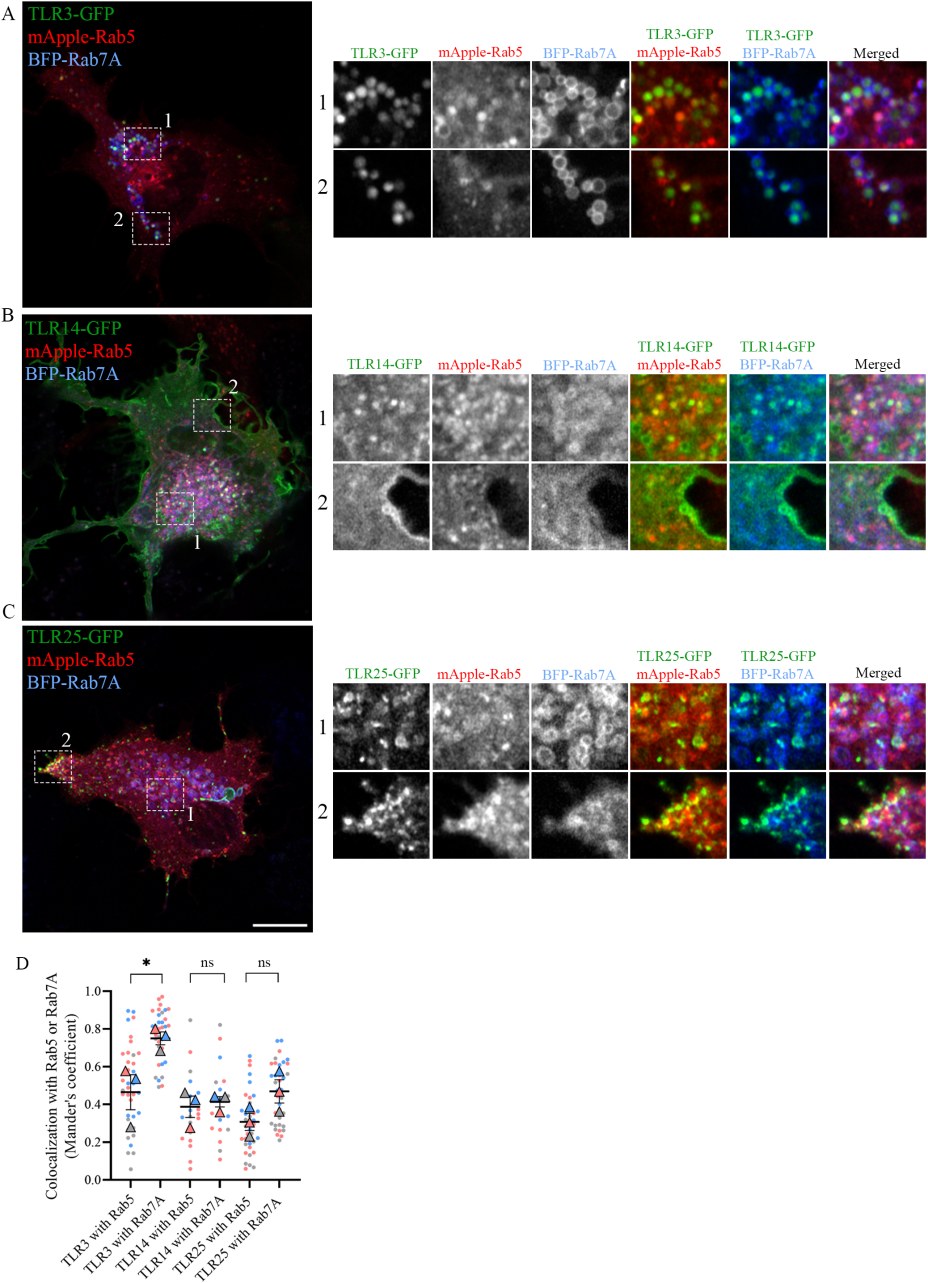
TLR3, TLR14 and TLR25 show different distribution along the endocytic pathway. Representative images of ACL cells transiently co-transfected with mCherry-Rab5 and BFP-Rab7A together with either Atlantic cod **(A)** TLR3-GFP, **(B)** TLR14-GFP, or **(C)** TLR25-GFP. Cells were imaged using a Zeiss LSM880 Fast AiryScan microscope. Scale bar: 10 µm. Magnification of boxed areas are shown to the right. **(D)** The graph represents colocalization (Manders’ coefficient) between Atlantic cod TLR3, TLR14, and TLR25 and either mCherry-Rab5 or BFP-Rab7A. Scatter plot shows the mean ± SEM from three independent experiments, color coded for each experimental repeat. Dots represent individual measurements and are color coded according to experimental repeat. n ≥ 19 cells in total. ns > 0.05, *p < 0.05 (two-tailed unpaired Student *t*-test).

To further dissect the localization of these Atlantic cod TLRs within the endocytic pathway, we next co-transfected them with the early endosomal marker mApple-Rab5 and the late endosomal marker BFP-Rab7A for super-resolution microscopy analysis. Image analysis revealed that TLR3-GFP localizes mainly to Rab7A-positive late endosomes (Mander’s coefficient 0.76), with a smaller fraction present also on early, Rab5-positive endosomes (Mander’s coefficient 0.41, **Figures 2A, D**). This is expected as TLR3 is known to locate to late endosomes in mammals (1). In addition to cell surface localization, TLR14-GFP displays equal distribution between early and late endosomes (Manders’ coefficient 0.41 for BFP-Rab7A, and 0.39 for mApple-Rab5, **Figures 2B, D**). TLR25-GFP localizes slightly more to late endosomes than early endosomes (Manders’ coefficient 0.47 for BFP-Rab7A, and 0.31 for mApple-Rab5, **Figures 2C, D**). However, as a small fraction of TLR14- and TLR25-positive endosomes did not colocalize with either Rab5 or Rab7A, we next investigated if these endosomes could be recycling endosomes. ACL cells were therefore co-transfected with the GFP-tagged TLRs and the recycling endosomal marker mCherry-Rab11. The results revealed low colocalization for the three TLRs with Rab11-positive recycling endosomes, with the highest value for TLR25-GFP (Mander’s coefficient 0.09 for TLR3-GFP, 0.16 for TLR14-GFP, and 0.25 for TLR25-GFP, **Supplementary Figure 1**).

**Figure 3:**
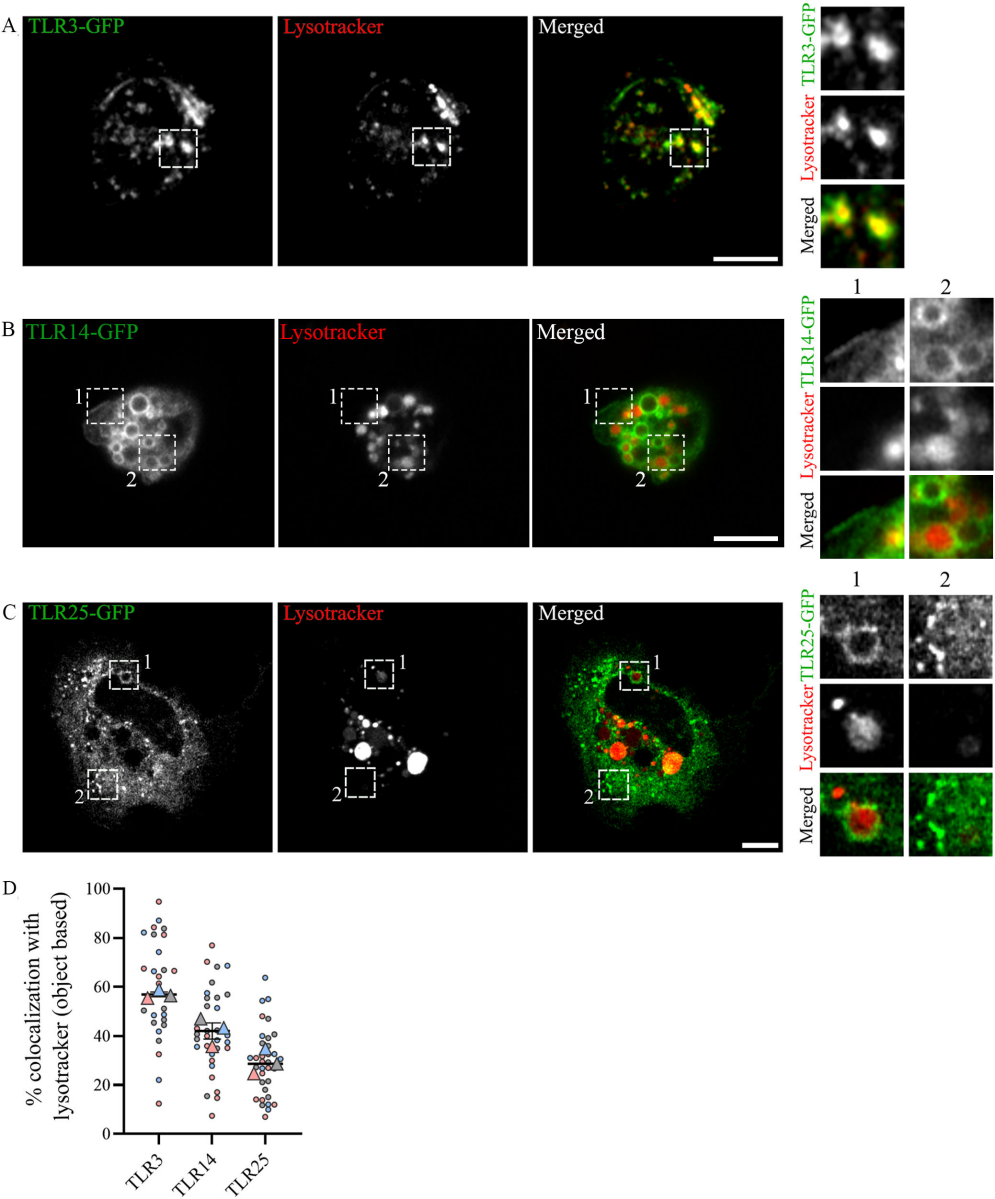
TLR3 localizes more to acidic compartments than TLR14 or TLR25 in primary leukocytes. Representative images of Atlantic cod primary leukocytes transiently transfected with either **(A)** TLR3-GFP, **(B)** TLR14-GFP, or **(C)** TLR25-GFP, stained using LysoTracker Red, then imaged using a Zeiss LSM880 Fast AiryScan microscope. Scale bar: 10 µm. Magnifications of boxed areas are shown to the right. The magnified image of TLR3 shows its location to perinuclear LysoTracker positive endosomes. The magnified images of TLR14 shows its location to LysoTracker positive endosomes (1) and the plasma membrane (2), whereas the magnified images of TLR25 show its perinuclear and LysoTracker positive endosomal location (1), or its location on small, peripheral endosomes negative for LysotTracker Red (2). **(D)** The graph represents colocalization (object-based) between TLR3, TLR14, or TLR25 and LysoTracker Red. Scatter plot shows the mean ± SEM from three independent experiments. Dots represent individual measurements, and the colours represent individual fish. n ≥ 27 cells in total.

We next verified the localization of the three TLRs in primary cells, transfecting both hepatocytes and leukocytes isolated from wild-caught Atlantic cod with GFP-tagged TLRs and then labelling the cells with LysoTracker Red. Consistent with the results from the ACL cell line, TLR3-GFP displayed the highest overlap with LysoTracker red compared to TLR14-GFP and TLR25-GFP in both leukocytes (**Figure 3**) and hepatocytes (**Supplementary Figure 2**). Moreover, TLR14-GFP also localized to the plasma membrane and TLR25-GFP to small, peripheral, LysoTracker Red-negative endosomes (**Figures 3B, C**; **Supplementary Figures 2B, C**) as in ACL cells. Co-transfection of TLRs with mApple-Rab7A followed by super-resolution microscopy confirmed similar colocalization in primary hepatocytes as in ACL cells (Mander’s coefficient for TLR3: 0.75, TLR14: 0.42, TLR25: 0.56, **Supplementary Figure 3**). The same trend of colocalization was also measured in primary leukocytes derived from wild-caught Atlantic cod (**Supplementary Figure 4**).

In sum, our results revealed that TLR14 is a plasma membrane protein as previously predicted (3), but it is also present on endosomes. Our results also indicate that TLR25 is an endosomal TLR in contrast to earlier suggestions indicating plasma membrane localization based on overall protein structure prediction and phylogenetic analysis (3).

### Atlantic cod TLR25 localization to small peripheral endosomes may depend on a dileucine-based cytosolic sorting signal

Even though Atlantic cod TLR25 has been proposed to be a cell surface TLR, a putative endolysosomal tyrosine-based sorting motif (YTKM) similar to the mammalian endosomal TLR3 and TLR9 sorting motif was identified in its cytosolic domain (3). As we revealed that TLR25 localizes to endosomes, we then investigated if the putative sorting motif is responsible for this localization. We therefore generated a TLR25 mutant lacking the putative cytosolic sorting signal by replacing the tyrosine residue (Y) in position 638 with an alanine residue (A), (**Supplementary Figure 5**). TLR25-GFP wild-type or TLR25-Y638A-GFP mutant were expressed in ACL cells and endolysosomes stained using LysoTracker Red. As we did not detect any difference in TLR25 localization to endolysosomes (**Supplementary Figures 5B–D**), we then investigated whether the putative sorting motif could be responsible for the TLR25 localization to recycling endosomes. To examine this, we performed Total Internal Reflection Fluorescence (TIRF) microscopy on ACL cells co-transfected with mApple-Rab11 and either TLR25-GFP wild-type or TLR25-Y638A-GFP. No statistically significant difference in colocalization was measured between the TLR25 wild-type or mutant and Rab11 (**Supplementary Figure 6**).

Altogether, these results indicate that the putative tyrosine-based sorting motif of TLR25 is not essential for the endosomal localization of TLR25 in Atlantic cod.

**Figure 4:**
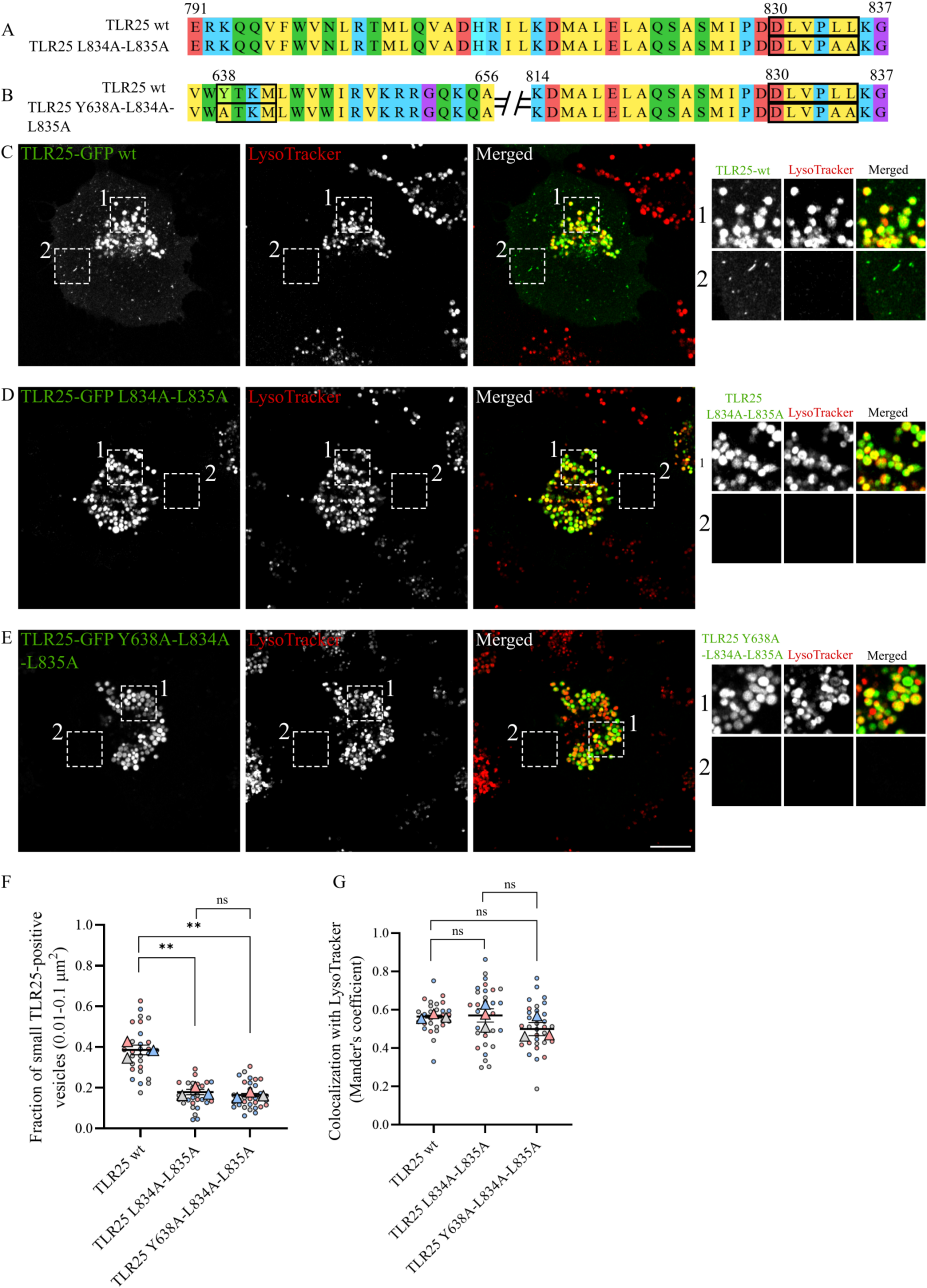
TLR25 localization to small peripheral vesicles may depend on a dileucine-based sorting signal in its C-terminal domain. **(A)** Part of the amino acid sequence of the Atlantic cod TLR25 wild-type C-terminal domain showing the putative endosomal sorting motif (DLVPLL) in the black box. The sequence below illustrates the point mutations L834A-L835A. **(B)** Part of the amino acid sequence of the Atlantic cod TLR25 wild-type cytosolic domain showing the putative endosomal sorting motifs (YTKM) and (DLVPLL) in the black boxes. The sequence below illustrates the point mutations introduced in the TLR25 mutant Y638A-L834A-L835A. Representative images of ACL cells transiently transfected with **(C)** TLR25-GFP wild-type, **(D)** TLR25-L834A-L835A-GFP, or **(E)** TLR25-Y638A-L834A-L835A-GFP, then stained with LysoTracker Red and imaged using a Zeiss LSM880 Fast AiryScan microscope. Scale bar: 10 µm. Magnification of boxed areas are shown to the right. **(F)** The graph represents the fraction of TLR25-positive vesicles with an area between 0.01 and 0.1 µm^2^. **(G)** The graph represents colocalization (Mander’s coefficient) with LysoTracker Red for TLR25 wild-type, TLR25-L834A-L835A, and TLR25-Y638A-L834A-L835A. **(F, G)** Scatter plots show the mean ± SEM from three independent experiments, and the colors represent individual repeats. n ≥ 28 cells in total. ns > 0.05, **p < 0.01 (two-tailed unpaired Student *t*-test).

We then identified a second potential sorting motif, dileucine-based (DLVPLL), near the C-terminus of TLR25. This sorting signal has previously been identified as a candidate sorting motif for MHC I (14). To investigate whether this motif is involved in TLR25-sorting to endosomes, two additional mutants were generated. In the first mutant, the two leucine residues (L) in position 834 and 835 were replaced with alanine residues (TLR25 L834A-L835A) (**Figure 4A**). In the second mutant, the previously described Y638A mutation was combined with the L834A-L835A mutation, disrupting both putative sorting motifs (**Figure 4B**). TLR25-GFP wild-type, TLR25-L834A-L835A-GFP, or TLR25-Y638A-L834A-L835A-GFP mutants were expressed in ACL cells, and endolysosomes were labelled using LysoTracker Red. Although there was no significant difference in colocalization between LysoTracker and TLR25 wild-type or either mutants, removal of the putative dileucine-based sorting motif caused over 50% reduction in the number of small peripheral TLR25-positive vesicles (**Figures 4C–G**). This suggests that the two leucines in position 834–835 may be part of a dileucine-based sorting signal in the cytosolic tail of TLR25 responsible for the transport of this receptor to the peripheral endosomes.

### Infection with *Francisella noatunensis* subsp. *noatunensis* results in perinuclear relocation of TLR25-positive endosomes

**Figure 5:**
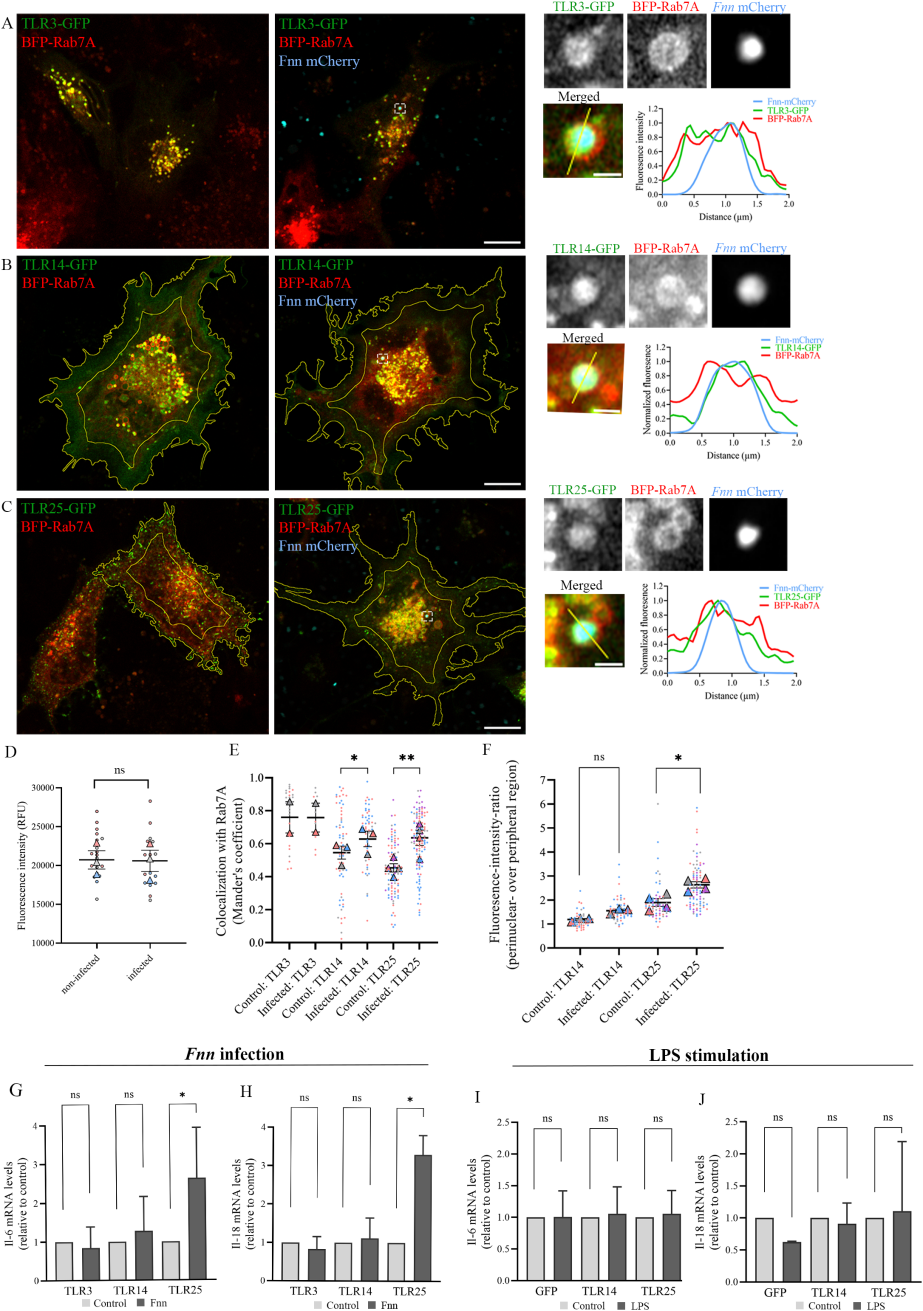
Atlantic cod TLR25 responds to *Francisella noatunensis* subsp. *noatunensis* (*Fnn*) infection. Representative images of ACL cells transiently co-transfected with BFP-Rab7A together with either **(A)** TLR3-GFP, **(B)** TLR14-GFP, or **(C)** TLR25-GFP. Cells were infected with mCherry-expressing *Fnn* and imaged using a Zeiss LSM880 Fast AiryScan microscope. Scale bar: 10 µm. Magnification of boxed areas are shown to the right with the yellow line indicating the line used for fluorescence intensity-profile analysis. Scale bar: 1 µm. **(D)** Graph represents raw fluorescent output at 530 nm excitation and 590 nm emission of non-infected vs *Fnn* infected ACL cells. Scatter plot shows the mean ± SEM from three independent experiments. Each experiment is an average of 6 measurements. **(E)** The graph represents colocalization (Mander’s coefficient) between TLR3-GFP, TLR14-GFP, or TLR25-GFP and BFP-Rab7A. colour n ≥ 13 cells in total. **(F)** The graph represents the ratio between the total fluorescence intensity from TLR14-GFP or TLR25-GFP in the peripheral region (outer yellow ring) over the perinuclear region (inner yellow ring). n ≥ 53 cells in total. **(E, F)** Scatter plots show the mean ± SEM from at least three independent experiments, color coded for each experimental repeat. Dots represent individual measurements and are color coded according to experimental repeats. Quantification of mRNA levels of IL-6 **(G)** and IL-18 **(H)** in control or *Fnn* infected ACL cells expressing either TLR3-GFP, TLR14-GFP, or TLR25-GFP. Quantification of mRNA levels of IL-6 **(I)** and IL-18 **(J)** in control or LPS stimulated ACL cells expressing either GFP, TLR14-GFP, or TLR25-GFP. **(G, H, I, J)** Levels of mRNA were normalized to the amount of 18s. Data represents the mean ± SD from at least three independent experiments. ns > 0.05, *p < 0.05, **p < 0.01 (two-tailed unpaired Student *t*-test).

TLRs are activated by binding to specific ligands, however the ligands for many teleost-specific TLRs remain unknown. To investigate if bacterial surface patterns, such as lipopolysaccharides (LPS), could be a potential ligand for TLR14 and TLR25, we infected ACL cells with the facultative intracellular Gram-negative bacterium *Francisella noatunensis* subsp. *noatunensis (Fnn)*, the causative agent of francisellosis in Atlantic cod (23). Since ACL cells do not have high phagocytic activity, a high multiplicity of infection (MOI) was required for sufficient bacterial internalization. To confirm that this did not induce significant cellular stress, an Alamar Blue viability assay was performed to measure metabolic activity during infection. No significant differences were observed between the infected and non-infected cells, indicating that the high bacterial load does not induce significant cellular stress and cell death (**Figure 5D**).

ACL cells were then co-transfected with GFP-tagged TLRs and BFP-Rab7A, then infected with mCherry-expressing *Fnn* for 6 hours before analysis by super-resolution microscopy. The results show that all TLRs colocalized with internalized *Fnn*-mCherry (**Figures 5A-C**). Interestingly, colocalization analysis between TLRs and BFP-Rab7A showed significant increase in colocalization between TLR14-GFP and BFP-Rab7A and even more between TLR25-GFP and BFP-Rab7A in *Fnn* infected cells as compared to uninfected cells (**Figure 5E**). Moreover, in *Fnn*-mCherry infected cells, TLR25-GFP, but not TLR14-GFP, showed a significant relocation from the peripheral to the perinuclear region (**Figure 5F**). This was also confirmed in primary leukocytes transfected with GFP-tagged TLRs and infected with *Fnn*-mCherry (**Supplementary Figures 7A–G**), suggesting that Atlantic cod TLR25 plays a role during *Fnn* infection.

### Infection with *Francisella noatunensis* subsp*. noatunensis* results in up-regulation of pro-inflammatory cytokines in cells overexpressing TLR25

Activation of TLRs results in up-regulation of genes such as pro-inflammatory cytokines (1). To further investigate the TLR-mediated response during *Fnn* infection, we next analyzed the expression of IL-6 and IL-18 by quantitative real-time PCR in ACL cells transiently transfected with GFP-tagged TLR3, TLR14, or TLR25 and either not infected or infected for 6 hours with *Fnn*. IL-6 and IL-18 were selected because of their key roles in inflammatory responses in both mammals and teleosts (24). Out of the three TLRs, only the cells overexpressing TLR25 showed significant up-regulation of IL-6 and IL-18 at 6 hours post-infection with *Fnn* as compared to uninfected cells (average fold-change 2.6 and 3.3, respectively) (**Figures 5G, H**). Similar results were observed in primary leukocytes, with significant up-regulation of IL-6 and IL-18 one hour after *Fnn* infection in cells overexpressing TLR25 (**Supplementary Figures H, I**). To investigate whether canonical Gram-negative surface patterns could act as ligands for TLR14 and TLR25, we stimulated ACL cells transfected with either TLR14-GFP or TLR25-GFP with LPS, an important outer membrane component of Gram-negative bacteria such as *Fnn*. LPS stimulation did not induce significant up-regulation of the pro-inflammatory cytokines IL-6 and IL-18 (**Figures 5I, J**), suggesting that LPS is not the ligand for either TLR14 or TLR25. Overall, the results from *Fnn* infection suggest that TLR25, but not TLR3 or TLR14, recognize *Fnn* bacterial structures different from LPS.

## Discussion

Atlantic cod has a unique TLR repertoire, lacking TLR1/6 and TLR2, members of the TLR1-family that recognize peptidoglycan and lipoproteins (3, 13). This implies that Atlantic cod has lost all homologs of known mammalian bacterial-recognizing surface TLRs (13). However, phylogenetic analysis suggests that it may potentially use TLR14 and TLR25, teleost-specific TLR1-family related members, to recognize peptidoglycan and lipoproteins (3). Therefore, in this study, we have investigated if TLR14 and TLR25 could potentially compensate for the lack of TLR1-family members.

TLR14 is a well conserved TLR in teleosts, lamprey, and amphibians with very little variation in terms of copy numbers (3, 17). We found TLR14 both at the plasma membrane and on endosomes, with equal distribution between early, Rab5-positive and late, Rab7A-positive endosomes. These findings support the hypothesis of TLR14 having TLR1-family-like function with plasma membrane localization for potential exposure and detection of peptidoglycan or lipopolysaccharides-like bacterial ligands (3). This is in line with previous studies in different fish species showing transcriptional up-regulation in response to a range of different stimuli such as Gram-negative bacteria (*Aeromonas hydrophila*, *Edwardsiella tarda*, *Photobacterium damselae*, and *Pseudomonas aeruginosa)*, Gram-positive bacteria (*Nocardia seriolae*), and by different ligands including LPS, LTA, flagellin, and Poly(I:C) (21, 25–30). TLR14 induction has also been reported during parasitic challenge (*Cryptocaryon irritans*) *(*31). The magnitude and timing of TLR14 responses vary among species, tissues, and time points, but overall, the literature supports its role as a broadly responsive receptor.

In contrast to mammalian surface TLRs, we also observed endosomal localization of Atlantic cod TLR14. This suggests that Atlantic cod TLR14 may recognize both bacterial surface components on the plasma membrane and pathogen-derived nucleic acids from within endosomes (**Supplementary Figure 9**), which is in agreement with the aforementioned studies. Similar observations have been described in a few other teleosts and lamprey where a portion of TLR14 is observed intracellularly, which also correlates with the wide range of ligands inducing up-regulation of transcription (21, 27, 32). Interestingly, one study in Japanese flounder reported interaction with both adaptor proteins MyD88 and TRIF, while lamprey TLR14 was shown to recruit MyD88 but not TRIF, indicating multiple possible downstream signaling pathways possibly due to different location (27, 32). This is a similar situation to what is observed for TLR4 in humans where ligand interaction induces signaling through both MyD88 and TRIF (33). However, the lack of response to *Fnn* in TLR14-overexpressing Atlantic cod cells, together with previous work showing that TLR14 is not differentially expressed in the head kidney of Atlantic cod infected with *Fnn (*34*)*, suggests that the specific ligand for Atlantic cod TLR14 is absent in *Fnn* and may not be present on the surface of Gram-negative bacteria. Nonetheless, as in other fish species TLR14 up-regulation in response to pathogens or ligands varies with ligand type, time-points and tissues investigated (21, 25, 27–29, 31), this could contribute to the lack of response observed in the present study.

For TLR25, a teleost specific TLR displaying significant copy number variation and also hypothesized to localize to the plasma membrane (3, 17), we found an exclusive endosomal localization, with higher localization on Rab7A-positive late endosomes than Rab5-positive early endosomes. This is in line with the intracellular localization of TLR25 from other fish species observed in other studies (35–37). Interestingly, the colocalization between TLR25-GFP and LysoTracker Red is lower than the colocalization between TLR25-GFP and mCherry-Rab7A. This is intriguing, as LysoTracker Red and Rab7A are both late endosomal markers and should therefore colocalize at a similar extent with TLR25. This effect is observed in both cell lines and primary cells, and is specific for TLR25, suggesting that Rab7A might be involved in the traffic of TLR25 to late endosomes. As we also observed significant relocation of TLR25-GFP to the perinuclear region upon *Fnn* infection, a dynein-mediated transport regulated by Rab7A (38), it is tempting to speculate that Rab7A regulates TLR25 traffic, and that this is further enhanced during *Fnn* infection. In line with this, the colocalization between TLR25-GFP and mApple-Rab7A increases in *Fnn* infected cells. Further supporting this hypothesis, previously reported transcriptomic data from Atlantic cod infected with *Fnn* showed transcriptional up-regulation of both Rab7A and TLR25 (34). Together, these results suggest that these two proteins might function together in responding to *Fnn* infection. The relocalization of TLR25-positive endosomes to the perinuclear region may imply fusion with lysosomes to facilitate degradation of the TLR25-bound ligand or downstream signaling from these endosomes.

TLR25’s localization to endosomes suggests that it detects nucleic acids from internalized pathogens. However, up-regulation of TLR25 in response to a range of different pathogens and ligands have been reported (*Aeromonas hydrophila*, *Edwardsiella ictaluri*, Grass carp reovirus, the parasite *Ichthyophthirius multifiliis*, and LPS), suggesting that it may recognize structures present on the surface of pathogens (22, 35, 36, 39–42). Nevertheless, these observations do not contradict each other as in this study we investigated only one of the nine TLR25 variants present in the Atlantic cod genome (13)recognize. Despite the sequences of the nine TLR25 variants being highly similar, differences are observed mainly in the extracellular, ligand-binding domain (3). These differences could therefore allow the different TLR25 variants to detect diverse PAMPs.

Among the TLRs investigated in this study, only TLR25 relocalized in Atlantic cod cells infected with *Fnn*. This suggests that it is the only TLR analyzed in the present work that is able to recognize patterns present on the facultative intracellular bacterium *Fnn*. In agreement with this, only cells overexpressing TLR25-GFP showed up-regulation of the pro-inflammatory cytokines IL-6 and IL-18 (43) after *Fnn* infection. Therefore, we suggest that TLR25 is triggered by *Fnn*, indicating a potential ligand specificity towards bacterial patterns. This is similar to other studies showing up-regulation of multiple inflammatory cytokines in other teleost species (35, 36, 44). Furthermore, gene expression analyses have shown that TLR25, but not TLR3 and TLR14, is up-regulated in Atlantic cod head kidney following *Fnn* infection, with up-regulation on day 2, 4, and 7 after infection. In the same study, IL-18 was also up-regulated on day 2, 4, and 7 after infection, with expression increasing over time (34). While IL-6 was not differentially expressed at these time points, we cannot exclude that it may be up-regulated at earlier time points as another work shows IL-6 up-regulation in macrophages during the first 12 hours of *Fnn* infection before its expression is down-regulated again (34, 45, 46). In mammals, IL-6 and IL-18 are also potent pro-inflammatory cytokines known to be up-regulated in response to bacterial infection (47–50). Therefore, up-regulation of these cytokines in response to *Fnn* in TLR25 but not TLR3 or TLR14 overexpressing cells further supports a potential ligand-specificity of TLR25 towards *Fnn*. The observed cytokine upregulation upon *Fnn* infection may partly reflect indirect effects of overexpression or altered endosomal trafficking. However, as we did not detect IL-6 and IL-18 upregulation in presence of LPS stimulation, this supports that the cytokine up-regulation upon *Fnn* infection is not an indirect effect of TLR overexpression.

Only IL-6 and IL-18 expression have been investigated in this study, and future works should include a broader range of cytokines to provide a more comprehensive understanding of the response triggered by *Fnn* infection in Atlantic cod.

Altogether, the function of TLR14 and TLR25 in Atlantic cod appears to be conserved regardless of MHC II loss, and our findings indicate that Atlantic cod does not rely solely on nucleic acid sensing TLRs. The phylogenetic relationships appear to be more correlated with function than overall predicted protein structure (3). A simultaneous study of other PRRs would be beneficial as there can be significant crosstalk between signaling pathways such as up-regulation of NFκB and through NOD-like and RIG-I like receptors (51). Future studies are also needed to characterize the specific ligands recognized by these and other Atlantic cod TLRs. The lack of cytokine up-regulation upon LPS stimulation in ACL cells suggests that Atlantic cod TLR14 and TLR25 do not recognize LPS, despite evidence that TLR14 may respond to LPS in other teleosts (32). Therefore, TLR25 may respond to other *Fnn*-derived PAMPs that remain unidentified. Potential candidates include other bacterial components, such as peptidoglycans. However, given the endosomal localization of TLR25, we cannot exclude the possibility that TLR25 responds to pathogen-derived nucleic acids. Furthermore, these findings are based on overexpression experiments and would ideally require additional validation through future loss-of-function studies.

In sum, in this work we have determined the intracellular localization of TLR3, TLR14, and TLR25 in Atlantic cod. We demonstrated that Atlantic cod is not completely devoid of cell surface TLRs as we detected TLR14 on the plasma membrane. Furthermore, we revealed that TLR25 resides on endosomes and responds to *Fnn* infection by inducing endosomal relocation and cytokine production (**Supplementary Figure 9**). Its endosomal localization suggests intracellular pathogen detection, including recognition of Gram-negative bacterial ligands.

## Materials and methods

### Cell culture

Atlantic cod larval (ACL) cells (52) were maintained in IMDM (Gibco) supplemented with 10% FCS, 2% Hepes buffer solution (Gibco), 1% L-glutamine (Gibco), 1% Non-Essential Amino Acids solution (Gibco), and 0.1% penicillin and streptomycin. Cells were kept in a 4.5% CO_2_ atmosphere at 15 °C.

### Sampling of fish

Atlantic cod were collected at a depth of 95–100 m in the inner Oslofjord (N 59°48.729, E 010°32.952) using demersal trawl between 2023 and 2026. Immediately after retrieval, the fish were euthanized by an acute blow to the head. Each individual was weighed and measured before blood was collected and hepatocytes and leukocytes isolated as described below. All personnel involved held ethical certification.

### Isolation of hepatocytes and leukocytes from Atlantic cod

Two-steps perfusion was done to isolate hepatocytes as previously described (53). In short, fish were opened from the ventral side and a cannula introduced in the largest hepatic vein. Following this vena cava was cut to allow perfusion of at least one lobe. Perfusion was done using perfusion buffer (NaCl 122 mM, KCl 4.8 mM, MgSO_4_ 1.2 mM, Na_2_HPO_4–_11 mM, NaH_2_PO_4_ 3.8 mM, and NaHCO_3_ 3.7 mM adjusted to pH 7.5). Two perfusion-steps were done, both for approximately 10–15 min (10 ml/min, adjusted with a speed regulator on the perfusion tube). In the first round, perfusion was done using perfusion buffer supplemented with ethylene glycol tetra acetic acid (EGTA, 26 μM) and done until all blood was replaced with buffer. In the second round, perfusion was done with perfusion buffer supplemented with 0.3 g/l collagenase type IV (Sigma) and 2 mM CaCl_2_ preheated to 37 °C for maximal collagenase activity. Afterwards, the perfused liver section was excised and suspended in ice-cold perfusion buffer supplemented with bovine serum albumin (BSA, 15 mM, Sigma) and cut into small pieces with a blade. The liver fragments were first filtered through a 200 μm cell strainer, and subsequently through a 100 μm cell strainer (pluriStrainer^®^, from Pluri Select), and then centrifuged three times at 50 × g. The pellet was first resuspended in ice-cold perfusion buffer containing BSA, then in Dulbecco’s Modified Eagle Medium (DMEM; Lonza, BioWhittaker) containing 5% fetal calf serum (FCS), 2 mM L-glutamine (Gibco), 100 U/mL penicillin, 100 μg/ml streptomycin, 0.25 µg/ml Amphotericin B (Sigma) and the last time, in DMEM containing 0.01 M Hepes (Gibco). Cells were plated into T25 culture flasks (VWR^®^) and stored at 4 °C until arrival at the lab where they were maintained at 10 °C with 4.5% CO_2_. Trypan blue (Sigma) was used to assess hepatocyte viability and cell density. Only samples with more than 70% viable hepatocytes were transfected. Transfection of hepatocytes was performed the same day as harvest.

Blood and head-kidney were used to isolate leukocytes from Atlantic cod as previously described (54). In brief, peripheral blood (1–2 ml) was collected from the caudal vein with a heparinized syringe (cannula 0.6 mm) and diluted 1:1.5 using cell culture medium (CCM) (Leibovitz L-15 (Sigma) supplemented with 5% FCS, 36 mM NaCl (380 mOsm), 10 U/ml Heparin, 0.29 mg/ml L-glutamine-, 100 U/ml Penicillin, 100 µg/ml Streptomycin, 0.25 µg/ml Amphotericin B, and 0.01 M Hepes). Head-kidney were excised from Atlantic cod and filtered through a 100 µm cell strainer (pluriStrainer^®^, from Pluri Select) using 1 ml ice cold CCM. The strainer was then rinsed with 4 ml of CCM. Isolation of leukocytes from peripheral blood and head-kidney cell suspension were then done by centrifugation on a discontinuous two-layered Percoll gradient (Cytiva) prepared in a 15 ml falcon tube containing 2.5 ml 1.070 g/l as bottom layer and a 2.0 ml 1.050 g/l as top layer. The samples were centrifuged at 400 × g at 10 °C for 40 min using a swing-out rotor. Leukocytes were collected from the interface between the 1.070 and 1.050 g/l layer and diluted 1:2 in CCM. At last, cells were centrifuged at 400 × g at 10 °C for 10 min and the pellet resuspended in 5 ml ice cold CCM. Isolated leukocytes were seeded into T25 culture flasks (VWR^®^) and stored at 4 °C until arrival at the lab where they were kept at 10 °C with 4.5% CO_2_. Leukocytes were transfected the day after harvest to allow proper cell adhesion, as only adherent cells were transfected.

### Sequence alignments and phylogenetic tree

All full-length TLR25 sequences from (3, 17) as well as TLR22 were used as queries in a blastX towards all gadmor3 protein gene models on www.ensembl.org (v115) in addition to Zebrafish and Spotted gar. The canonical transcript of all hits was downloaded and added to the queries in a protein multiple sequence alignment using MEGAX. A phylogenetic tree was inferred using the Neighbor-Joining method with Poisson correction, pairwise deletion and 500 bootstrap replicates. The multiple sequence alignment was slightly trimmed in the leading and tailing ends to remove variation due to inaccurate gene models resulting in e.g. missing start codon or significantly longer cytoplasmic tail. Then overall mean distance for TLR25 and TLR22 respectively was calculated as the mean number of amino acid differences across all sequences of the entire protein, but also for the ectodomain (ECTO), the transmembrane domain (TM) and the cytoplasmic domain (CYTO).

### Constructs and reagents

pEGFP-N3 constructs containing Atlantic cod TLR3, TLR14 and TLR25 were purchased from GenScript. Sequences are available at www.doi.org/10.6084/m9.figshare.25218863. TLR sequences are derived from (3), but updated to the newest available genome assembly from Atlantic cod (GCF_902167405.1) by BlastN. TLR25 construct is based on ENSGMOG00000036237, the TLR14 construct is based on ENSGMOG00000003793 and TLR3 is based on ENSGMOG00000035330. The rest of the constructs used in this study are of mammalian origin. Before expressing the mammalian constructs in cells derived from Atlantic cod, alignment analysis between mammalian and Atlantic cod endosomal and lysosomal markers were performed using their overall sequence and phylogenetic clustering (55, 56) with molecular markers showing high degree of conserveness.

The TLR25-mutants were obtained using the Quick Change II XL Site-Directed Mutagenesis Kit from Agilent Technologies using the pEGFP-N3-TLR25 as template. forwardPrimers were designed using QuickChange Primer Design (agilent.com) and purchased from Eurofins Genomics (**Table 1**).

**Table 1:** Primer sequences used for site-directed mutagenesis.

Primer		Sequence (5’-3’)
TLR25-Y638A-GFP	For	ctgtgacggagtgtgggccaccaagatgctgtgg
	Rev	ccacagcatcttggtggcccacactccgtcacag
TLR25-L834A-L835A-GFP	For	cccagacgatttggttcctgcggcaaaaggctccaccggctcc
	Rev	ggagccggtggagccttttgccgcaggaaccaaatcgtctggg

We used the protocol provided by the kit and 12 minutes extension time and 30 ng of dsDNA template. BFP-Rab7A was a kind gift from Yael Elbaz-Alon (Weizmann Institute of Science, Israel) (57). mApple-Rab7A (Addgene plasmid #54945; http://n2t.net/addgene:54945; RRID: Addgene_54945), and mApple-Rab11A (Addgene plasmid #54942; http://n2t.net/addgene:54942; RRID: Addgene_54942) were gifts from Michael Davidson. mCherry-Rab5A has been described before (58). LysoTracker™ Red DND-99 (Invitrogen) was used at a final concentration of 50 nM. Cells were incubated with LysoTracker for 15 min before imaging.

### Transfection

ACL cells and primary cells were transfected using Amaxa nucleofector 2b (Lonza), using the program X-005 and seeded onto 35-mm-diameter imaging dishes with glass bottom (MatTek) coated with 20 μg/μL fibronectin (Sigma). 1/6 of a confluent T75 (VWR^®^) flask was used for each transfection-reaction of ACL cells, while 10 (6) primary cells were used for each transfection-reaction. 2 µg DNA were used for each transfection. Cells were transfected for at least 4 days (for single transfection) or 7 days (for double/triple transfections) prior to downstream applications.

### Bacterial strain and growth conditions

The NCIMB14265 strain of *F. noatunensis* subsp*. noatunensis* (*Fnn*), isolated from diseased Atlantic cod (*Gadus morhua*) and transformed with the mCherry-expressing plasmid pKK289Km/mCherry (*Fnn-*mCherry) (59) was a kind gift from Hanne C. Winther-Larsen from the Department of Pharmacy, University of Oslo, Norway. *Fnn* stocks were maintained at -80 °C for long-term storage in BD Bacto Eugon broth (BD Diagnostic Systems) supplemented with 20% glycerol (Sigma). Before infection experiments, bacteria were seeded onto Eugon blood agar (EBA) plates (30.4 g/l BD Bacto Eugon, 15 g/l agar, 2 mM FeCl_3_ (Sigma), and 5% bovine blood, from Håtunalab), supplemented with 25 µg/ml kanamycin (Sigma) and incubated at 20 °C for 6–8 days until growth was observed on the plate. 3–4 days prior to infection, *Fnn* was inoculated in Eugon broth supplemented with 2 mM FeCl_3_ and 25 µg/ml kanamycin, incubated at 20 °C with shaking at 150 rpm for 2–4 days until bacteria reached an OD600 between 1.5-1.8.

### Infection of ACL cells and primary leukocytes

ACL cells were transfected and seeded either onto 24-well plates with glass bottom (MatTek) coated with 20 μg/μl fibronectin (Sigma) for microscopy analysis, or onto 6-well plates for gene expression analysis. Prior to infection, cells were washed 3 times in PBS, then incubated in IMDM medium without antibiotics and with heat-inactivated FCS. At day 7 after transfection, cells were infected with *Fnn*-mCherry growing in their exponential phase (OD_600_ = 1.5-1.8) and diluted to OD_600_ = 1 in IMDM medium without antibiotics and with heat-inactivated FCS. To synchronize bacterial uptake, ACL cells together with *Fnn* were centrifuged at 500 × g for 5 min. ACL cells were infected for 6 h with multiplicity of infection (MOI) of 2500 and kept at 15 °C, 4.5% CO_2_.

Atlantic cod primary leukocytes were transfected and seeded onto 24-well plates with glass bottom (MatTek) coated with 20 μg/μl fibronectin (Sigma) for microscopy analysis, or onto 6-well plates for gene expression analysis. Prior to infection, cells were washed 3 times in PBS, then incubated in supplemented Leibovitz L-15 medium without antibiotics and with heat-inactivated FCS. 3–4 days post transfection, cells were infected with *Fnn-*mCherry growing in their exponential phase (OD_600_ = 1.5-1.8) and diluted to OD_600_ = 0.5 in supplemented Leibovitz L-15 medium without antibiotics and with heat-inactivated FCS. The primary leukocytes together with *Fnn* were centrifuged at 500 x g for 5 min at 10 °C. Primary leukocytes were infected for 1 h with multiplicity of infection (MOI) of 500 and kept at 10 °C, 4.5% CO_2_.

### Cell viability assay

Cell viability of ACL cells after infection was measured using Alamar Blue, a resazurin-based solution that living cells can reduce to the fluorescent dye resorufin. The fluorescence intensity is proportional to the number of metabolically active cells (60). Approximately 50–000 ACL cells were seeded onto 96-well plates and incubated for 6–7 days until they reached 90-100% confluency. The ACL cells were infected as described above for 6 h with MOI of 2500 at 15 °C, 4.5% CO_2_. The cells were then washed 5 times in PBS 1 x to remove non internalized bacteria and incubated with 0.29 x Alamar Blue™ Cell Viability Reagent (Invitrogen) diluted in 50 mM Tris(hydroxymethyl)aminomethane for 30 minutes. Fluorescence was quantified at 530 nm excitation and 590 nm emission using a BioTek™ Synergy MX Microplate reader.

### Quantitative real-time RT-PCR

Total RNA was extracted from ACL cells infected with *Fnn* using RNeasy^®^ Plus Mini Kit (Qiagen) following the manufacturer’s protocol, including the recommended on-column DNase treatment. cDNA was produced from 1 µg RNA using the Transcriptor First Strand cDNA Synthesis Kit (Roche) with random hexamer primers. Real-time quantitative RT-PCR was done to amplify and quantify cDNA using the LightCycler^®^ 480 SYBR^®^ Green I Master mix (Roche) with the LightCycler^®^ 480 PCR system (Roche). Primers were purchased from Eurofins and are listed in **Table 2**. The PCR program used was: 1 cycle 10 min at 96 °C; 50 cycles 10 s at 95 °C, 10 s at 60 °C, 10 s at 72 °C; 1 cycle at 10 s at 95 °C, 60 s at 65 °C, and 1 sec 97 °C. Specificity and identity of PCR products were determined by performing melting curve tests. The transcript levels of 18s were used to normalize the samples and used as it has previously been described as house-keeping gene in gene expression analysis done in Atlantic cod (61).

**Table 2:** Primer sequences used for RT-qPCR.

Primer		Sequence (5’-3’)
IL-6	For	AATGCCCTTCTACCGCACAT
	Rev	CTCCGGGTGCCTCATCTTTT
IL-18	For	TACAAGGTGGTGTCCTGTCG
	Rev	TAGTCCCTTGTTCCGGTTCG
18s	For	CACACGGCTGGAAAAGTGAA
	Rev	GCGCGGGTTTTAAGTCTGAT

### Lipopolysaccharide stimulation of ACL cells

ACL cells were transfected and seeded onto 6-well plates for 6–7 days. The cells were washed 3 times in PBS, then incubated in 20 μg/μl lipopolysaccharide (LPS) from *Escherichia coli* O111:B4 (Sigma) diluted in IMDM medium without antibiotics and with heat-inactivated FCS. The cells were incubated for 6 h at 15 °C, 4.5% CO_2_, and lysed with RLT buffer from the RNeasy^®^ Plus Mini Kit (Qiagen) with 2% Dithiothreitol (Sigma). Quantitative real-time RT-PCR was performed as described above.

### Live-cell microscopy

Live-cell imaging was conducted at room temperature with 4.5% CO_2_. Imaging was done using a Zeiss LSM880 microscope equipped with a 63 × oil Plan Apo NA 1.4 objective, an Olympus SpinSR SoRA spinning disk confocal using a 60 × Planpon NA 1.42 objective, or a Leica Total Internal Reflection Fluorescence (TIRF) microscope using a 100 × oil HCK plan Apo 1.47 NA objective.

### Imaging processing and analysis

ImageJ (National Institutes of Health) and Adobe Photoshop (Adobe Systems) were used for image analysis and processing. GraphPad Prism 9 (GraphPad Software Inc., https://www.graphpad.com) was used for generating graphs. IMARIS analysis software (Oxford instruments) was used to create 3D reconstructions of z-stack microscopy images. Colocalization-analysis was done using pixel-based analysis for ACL cells, and object-based analysis for primary cells due to their higher degree of background noise. The pixel-based analysis method used Mander’s coefficient in the JACoP plugin in ImageJ (62). Object-based colocalization analysis was performed using ImageJ software to quantify the degree of overlap between the TLRs and other markers. The area corresponding to the TLR signal with an overlapping signal from LysoTracker or Rab7 was measured with respect to the total area of the TLR within the cell. The size of the TLR25-positive vesicles was quantified in ImageJ by generating a binary mask. The ratio was calculated as the number of TLR25-positive vesicles with an area between 0.01 and 0.1µm^2^ divided by the total number of TLR25-positive vesicles. Fluorescence intensity profile analysis was obtained using the plot profile function in ImageJ. Total emitted fluorescence-analysis between perinuclear and peripheral region was done in ImageJ by measuring the RawIntDen/area from each region. The perinuclear and peripheral regions were defined by creating an ROI of the cell-outline, then a smaller section was created by using the “enlarge selection-function imputing negative values” creating a smaller section having half the area of the large section. While the inner ROI represents the perinuclear region, the peripheral region is the region between the inner ROI and the cell-outline. The ratio between the two sections was measured by dividing the RawIntDen/area from the peripheral region over the RawIntDen/area from the perinuclear region. Statistical analysis was done using two-tailed unpaired Student’s *t*-test as two conditions were compared to each other, in Excel (Microsoft). In the figures, statistical significance is indicated as follows: ns > 0.05, *p < 0.05, **p < 0.01. Schematic illustration was generated using BioRender (BioRender.com).

## Data Availability

Sequences are available at www.doi.org/10.6084/m9.figshare.25218863.

## Supplementary material

Supplementary Figure 1 TLR25 localize more than TLR14 and TLR3 to recycling endosomes. **(A)** The number of amino acid differences per sequence from averaging over all sequence pairs are shown. The results were calculated first across the different functional domains (ectodomain (Ecto), transmembrane domain (Tm), and cytoplasmic tail (Cyto)) and then for the complete sequence (All). All positions with less than 95% site coverage were eliminated. Analysis conducted in MEGA X - compute overall mean differences function. Representative images of ACL cells transiently co-transfected with mCherry-Rab11 and **(B)** TLR3-GFP, **(C)** TLR14-GFP, or **(D)** TLR25-GFP and imaged using a Zeiss LSM880 Fast AiryScan microscope. Scale bar: 10 µm. Magnification of boxed areas are shown to the right. **(E)** The graph represents colocalization (Manders’ coefficient) between TLR3-GFP, TLR14-GFP, or TLR25-GFP and mCherry-Rab11. Scatter plot shows the mean ± SEM from three independent experiments. Dots represent individual measurements and are color coded for each experimental repeat. n ≥ 31 cells in total.

Supplementary Figure 2 TLR3 localizes more to acidic compartments than TLR14 or TLR25 in primary hepatocytes. Representative images of Atlantic cod primary hepatocytes transiently transfected with either (**A**) TLR3-GFP, (**B**) TLR14-GFP, or (**C**) TLR25-GFP, stained using LysoTracker Red, and imaged using an Olympus SpinSR SoRA microscope. Scale bar: 10 µm. Magnification of boxed areas are shown to the right. The magnified image of TLR14 shows its location to the plasma membrane and to endosomes, whereas the magnified images of TLR25 show its perinuclear and LysoTracker positive endosomal location (1), or its location on small, peripheral endosomes negative for LysoTracker Red (2). (**D**) The graph represents colocalization (Manders’ coefficient) between TLR3, TLR14, or TLR25 and LysoTracker Red. Scatter plot shows the mean ± SEM from n ≥ 16 cells in total from two fish.

Supplementary Figure 3 In primary hepatocytes, TLR3, TLR14, and TLR25 colocalize with Rab7A at the same extent as in ACL cells. Representative images of Atlantic cod primary hepatocytes transiently co-transfected with mApple-Rab7A together with either **(A)** TLR3-GFP, **(B)** TLR14-GFP, or **(C)** TLR25-GFP and imaged using an Olympus SpinSR SoRA microscope. Scale bar: 10 µm. Magnification of boxed areas are shown to the right. **(D)** The graph represents colocalization (Manders’ coefficient) between TLR3, TLR14, or TLR25 and mApple-Rab7A. Scatter plot shows the mean ± SEM from n ≥ 21 cells in total from two fish.

Supplementary Figure 4 In primary leukocytes, TLR3, TLR14 and TLR25 colocalize with Rab7A at the same extent as in ACL cells. Representative images of Atlantic cod primary leukocytes transiently co-transfected with mApple-Rab7A and **(A)** TLR3-GFP, **(B)** TLR14-GFP, or **(C)** TLR25-GFP and imaged using a Zeiss LSM880 Fast AiryScan microscope. Scale bar: 5 µm. Magnification of boxed areas are shown to the right. **(D)** The graph represents the percentage of overlap between TLR3, TLR14, or TLR25 and mApple-Rab7A. Colocalization was analyzed using object-based colocalization analysis in ImageJ. Scatter plot shows the mean ± SEM from three independent experiments. Dots represent individual measurements, and the colors represent individual fish. n ≥ 31 cells in total.

Supplementary Figure 5 TLR25 localizes to endosomes independently of the predicted tyrosine-based cytosolic sorting signal. **(A)** Part of the amino acid sequence of the Atlantic cod TLR25 wild-type cytosolic domain showing the putative endosomal sorting motif (YTKM) in the black box. The sequence below illustrates the point mutation Y638A. Representative images of ACL cells transiently transfected with **(B)** TLR25-GFP wild-type or **(C)** TLR25-Y638A-GFP, then stained with LysoTracker Red and imaged using a Zeiss LSM880 Fast AiryScan microscope. Scale bar: 10 µm. Magnification of boxed areas are shown to the right. **(D)** The graph represents colocalization (Manders’ coefficient) with LysoTracker Red for TLR25 wild-type and TLR25-Y638A. Scatter plot shows the mean ± SEM from three independent experiments, and the colors represent individual repeats. n ≥ 36 cells in total. ns > 0.05 (two-tailed unpaired Student *t*-test).

Supplementary Figure 6 TLR25 localizes to recycling endosomes independently of the predicted cytosolic sorting signal. Representative images of ACL cells transiently co-transfected with mApple-Rab11 and **(A)** TLR25-GFP wild-type or **(B)** TLR25-Y638A-GFP and imaged using a Leica Total Internal Reflection Fluorescence (TIRF) microscope with an optical section of 100 nm. Scale bar: 10 µm. Magnification of boxed areas are shown to the right. **(C)** The graph represents colocalization (Manders’ coefficient) between TLR25 wild-type or TLR25-Y638A and mApple-Rab11. Scatter plot shows the mean ± SEM from three independent experiments, color coded for each experimental repeat. n ≥ 40 cells in total.

Supplementary Figure 7 Atlantic cod TLR25 responds to *Francisella noatunensis* subsp. *noatunensis* (*Fnn*) infection in primary leukocytes. Representative images of Atlantic cod primary leukocytes imaged using a Zeiss LSM880 Fast AiryScan microscope. Scale bars: 5 µm. The cells were transfected with TLR3-GFP and either non-infected **(A)** or infected with mCherry-expressing *Fnn* **(B),** transfected with TLR14-GFP and non-infected **(C)** or infected with mCherry-*Fnn* **(D)** or transfected with TLR25-GFP and non-infected **(E)** or infected with mCherry-*Fnn* **(F)**. Magnification of boxed areas are shown to the right as IMARIS isosurface 3D rendering of a TLR-positive vesicle containing *Fnn*-mCherry. **(G)** The graph represents the ratio between the total fluorescence intensity from TLR3-GFP, TLR14-GFP, or TLR25-GFP in the perinuclear region over the peripheral region in non-infected vs. infected cells. Scatter plot shows the mean ± SEM from 5 fish per condition. Dots represent individual measurements, and the colors represent individual fish. n ≥ 52 cells in total. Quantification of mRNA levels of IL-6 **(H)** and IL-18 **(I)** in control or *Fnn* infected primary leukocytes expressing either TLR3-GFP, TLR14-GFP, or TLR25-GFP. Levels of mRNA were normalized to the amount of 18s and to the levels of cytokines in infected leukocytes expressing only GFP. Data represents the mean ± SD from three fish (TLR14 and TLR25) and one fish (TLR3). ns > 0.05, *p < 0.05, **p < 0.01 (two-tailed unpaired Student *t*-test).

Supplementary Figure 8 Phylogenetic tree displayed as cladogram for TLR3, TLR14, TLR22 and TLR25 sequences manually annotated in the reference genome gadmor3 with available gene models from Ensembl.org. Sequences forming the base of constructs highlighted in blue. The tree was inferred using the Neighbor-Joining method, poisson distribution and pairwise deletion on a protein sequence alignment (ClustalW) using MEGAX. The most similar gene models for each construct are TLR3 ENSGMOG00000035330, TLR14 ENSGMOG00000003793 and TLR25 ENSGMOG00000036237.

Supplementary Figure 9 Schematic overview of proposed TLR trafficking. TLR3 localizes to endosomes and is predicted to recognize dsRNA, similar to its mammalian ortholog. TLR14 localizes to both endosomes and the plasma membrane and may recognize nucleic acids and bacterial surface components in these compartments, respectively. TLR25 localizes to endosomes and small peripheral endosomes and responds to *Fnn* infection by relocalizing to the perinuclear region of the cell. Created in BioRender.com. Krokene, P. (2026).

Supplementary Video 1 TLR14 is present on the plasma membrane and is internalized onto endosomes. ACL cells transiently transfected with TLR14-GFP and imaged using a Zeiss LSM880 Fast AiryScan microscope. Scale bar: 10 µm. The boxed area indicates the section included in **Figure 1E**. Images acquired every 10 seconds.
