# Supplementary figures and images for: TLR25 is endosomally located and responds to Francisella infection in Atlantic cod
Source: Front Immunol. 2026 Jul 8;17:1798290. doi: 10.3389/fimmu.2026.1798290 (PMC13388067; doi:10.3389/fimmu.2026.1798290)

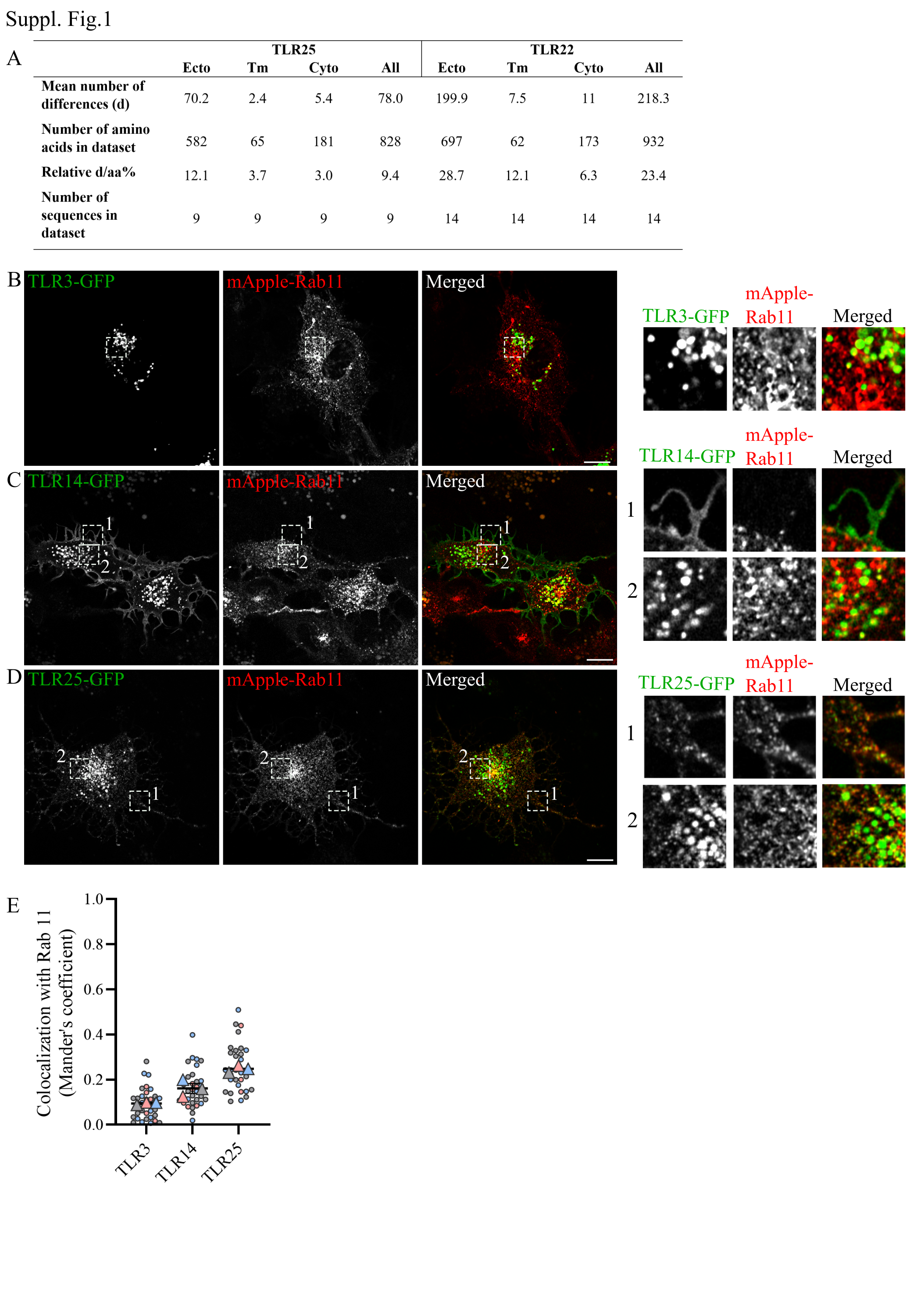

Supplement: Supplementary Figure 1 — TLR25 localize more than TLR14 and TLR3 to recycling endosomes. (A) The number of amino acid differences per sequence from averaging over all sequence pairs are shown. The results were calculated first across the different functional domains (ectodomain (Ecto), transmembrane domain (Tm), and cytoplasmic tail (Cyto)) and then for the complete sequence (All). All positions with less than 95% site coverage were eliminated. Analysis conducted in MEGA X - compute overall mean differences function. Representative images of ACL cells transiently co-transfected with mCherry-Rab11 and (B) TLR3-GFP, (C) TLR14-GFP, or (D) TLR25-GFP and imaged using a Zeiss LSM880 Fast AiryScan microscope. Scale bar: 10 µm. Magnification of boxed areas are shown to the right. (E) The graph represents colocalization (Manders’ coefficient) between TLR3-GFP, TLR14-GFP, or TLR25-GFP and mCherry-Rab11. Scatter plot shows the mean ± SEM from three independent experiments. Dots represent individual measurements and are color coded for each experimental repeat. n ≥ 31 cells in total. [file Image1.tiff]

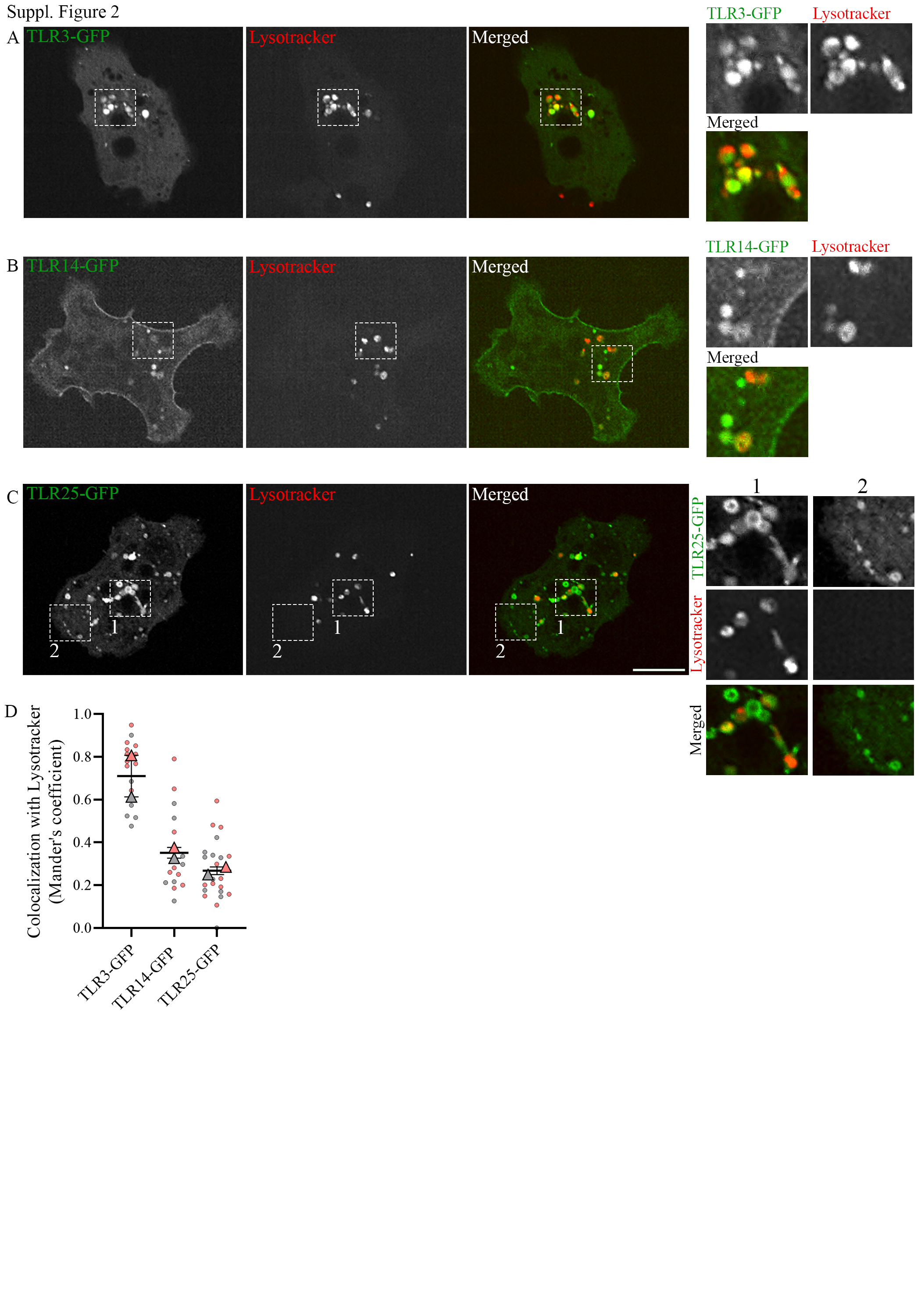

Supplement: Supplementary Figure 2 — TLR3 localizes more to acidic compartments than TLR14 or TLR25 in primary hepatocytes. Representative images of Atlantic cod primary hepatocytes transiently transfected with either (A) TLR3-GFP, (B) TLR14-GFP, or (C) TLR25-GFP, stained using LysoTracker Red, and imaged using an Olympus SpinSR SoRA microscope. Scale bar: 10 µm. Magnification of boxed areas are shown to the right. The magnified image of TLR14 shows its location to the plasma membrane and to endosomes, whereas the magnified images of TLR25 show its perinuclear and LysoTracker positive endosomal location (1), or its location on small, peripheral endosomes negative for LysoTracker Red (2). (D) The graph represents colocalization (Manders’ coefficient) between TLR3, TLR14, or TLR25 and LysoTracker Red. Scatter plot shows the mean ± SEM from n ≥ 16 cells in total from two fish. [file Image2.tif]

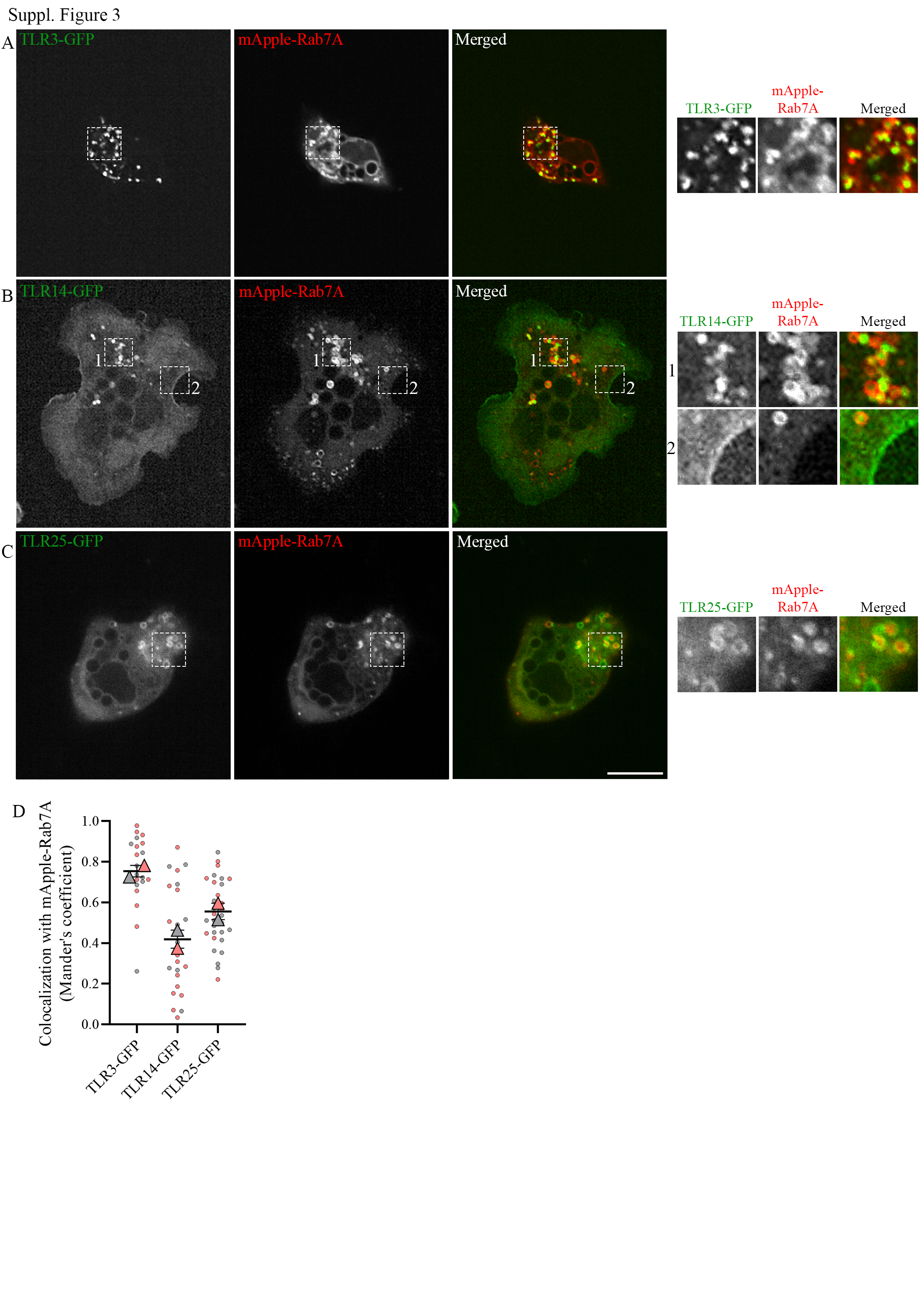

Supplement: Supplementary Figure 3 — In primary hepatocytes, TLR3, TLR14, and TLR25 colocalize with Rab7A at the same extent as in ACL cells. Representative images of Atlantic cod primary hepatocytes transiently co-transfected with mApple-Rab7A together with either (A) TLR3-GFP, (B) TLR14-GFP, or (C) TLR25-GFP and imaged using an Olympus SpinSR SoRA microscope. Scale bar: 10 µm. Magnification of boxed areas are shown to the right. (D) The graph represents colocalization (Manders’ coefficient) between TLR3, TLR14, or TLR25 and mApple-Rab7A. Scatter plot shows the mean ± SEM from n ≥ 21 cells in total from two fish. [file Image3.tif]

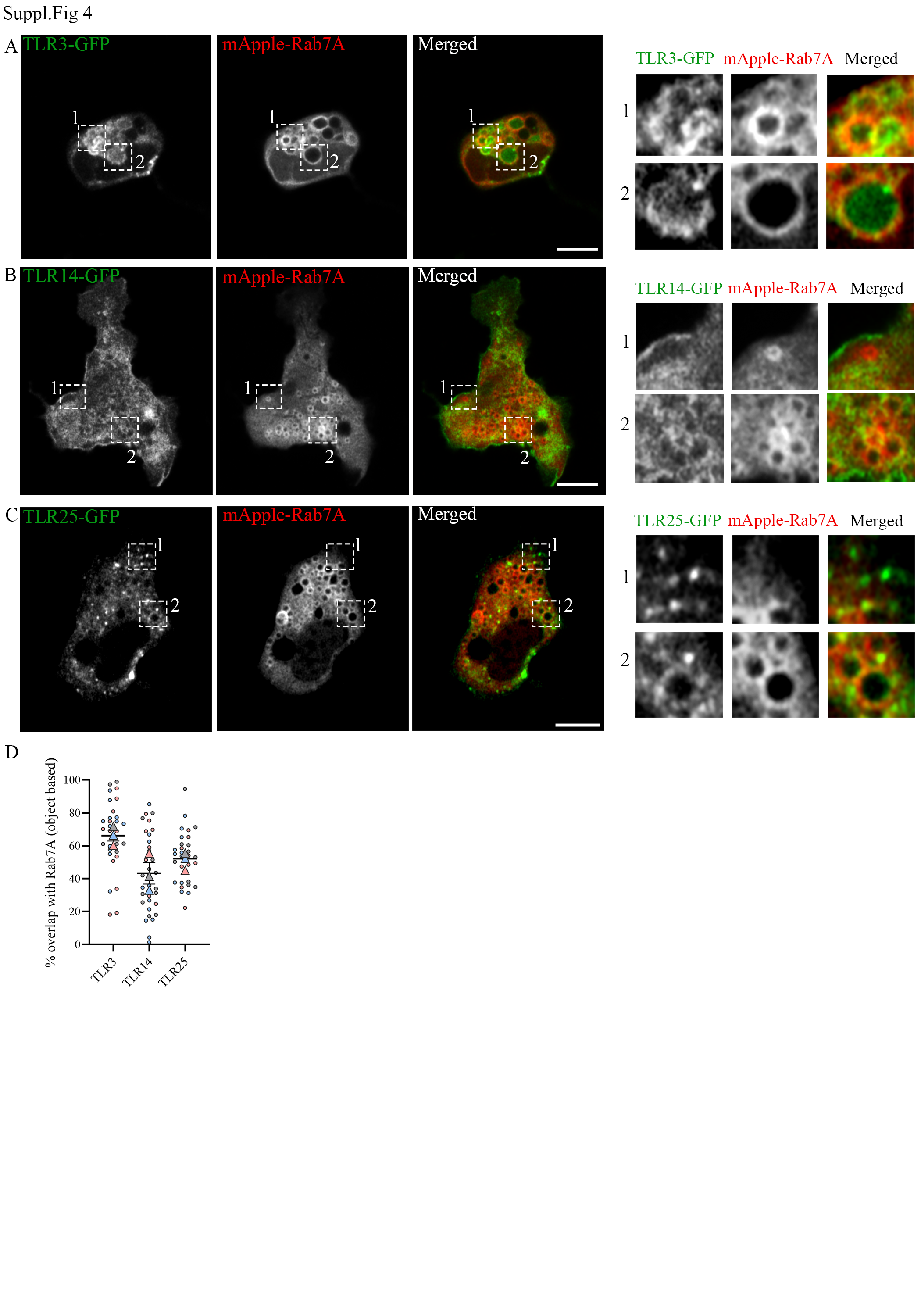

Supplement: Supplementary Figure 4 — In primary leukocytes, TLR3, TLR14 and TLR25 colocalize with Rab7A at the same extent as in ACL cells. Representative images of Atlantic cod primary leukocytes transiently co-transfected with mApple-Rab7A and (A) TLR3-GFP, (B) TLR14-GFP, or (C) TLR25-GFP and imaged using a Zeiss LSM880 Fast AiryScan microscope. Scale bar: 5 µm. Magnification of boxed areas are shown to the right. (D) The graph represents the percentage of overlap between TLR3, TLR14, or TLR25 and mApple-Rab7A. Colocalization was analyzed using object-based colocalization analysis in ImageJ. Scatter plot shows the mean ± SEM from three independent experiments. Dots represent individual measurements, and the colors represent individual fish. n ≥ 31 cells in total. [file Image4.tif]

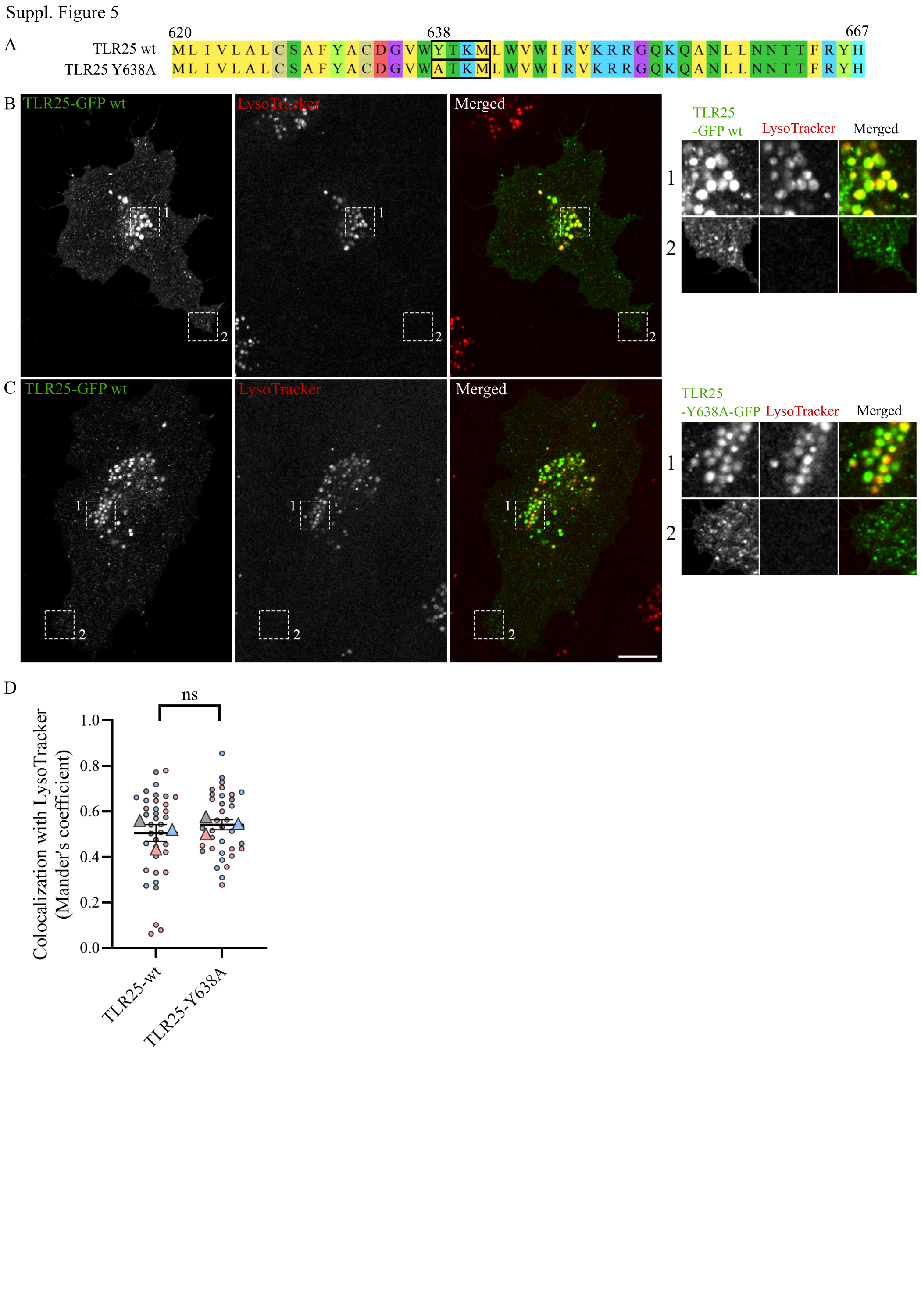

Supplement: Supplementary Figure 5 — TLR25 localizes to endosomes independently of the predicted tyrosine-based cytosolic sorting signal. (A) Part of the amino acid sequence of the Atlantic cod TLR25 wild-type cytosolic domain showing the putative endosomal sorting motif (YTKM) in the black box. The sequence below illustrates the point mutation Y638A. Representative images of ACL cells transiently transfected with (B) TLR25-GFP wild-type or (C) TLR25-Y638A-GFP, then stained with LysoTracker Red and imaged using a Zeiss LSM880 Fast AiryScan microscope. Scale bar: 10 µm. Magnification of boxed areas are shown to the right. (D) The graph represents colocalization (Manders’ coefficient) with LysoTracker Red for TLR25 wild-type and TLR25-Y638A. Scatter plot shows the mean ± SEM from three independent experiments, and the colors represent individual repeats. n ≥ 36 cells in total. ns > 0.05 (two-tailed unpaired Student t-test). [file Image5.tiff]

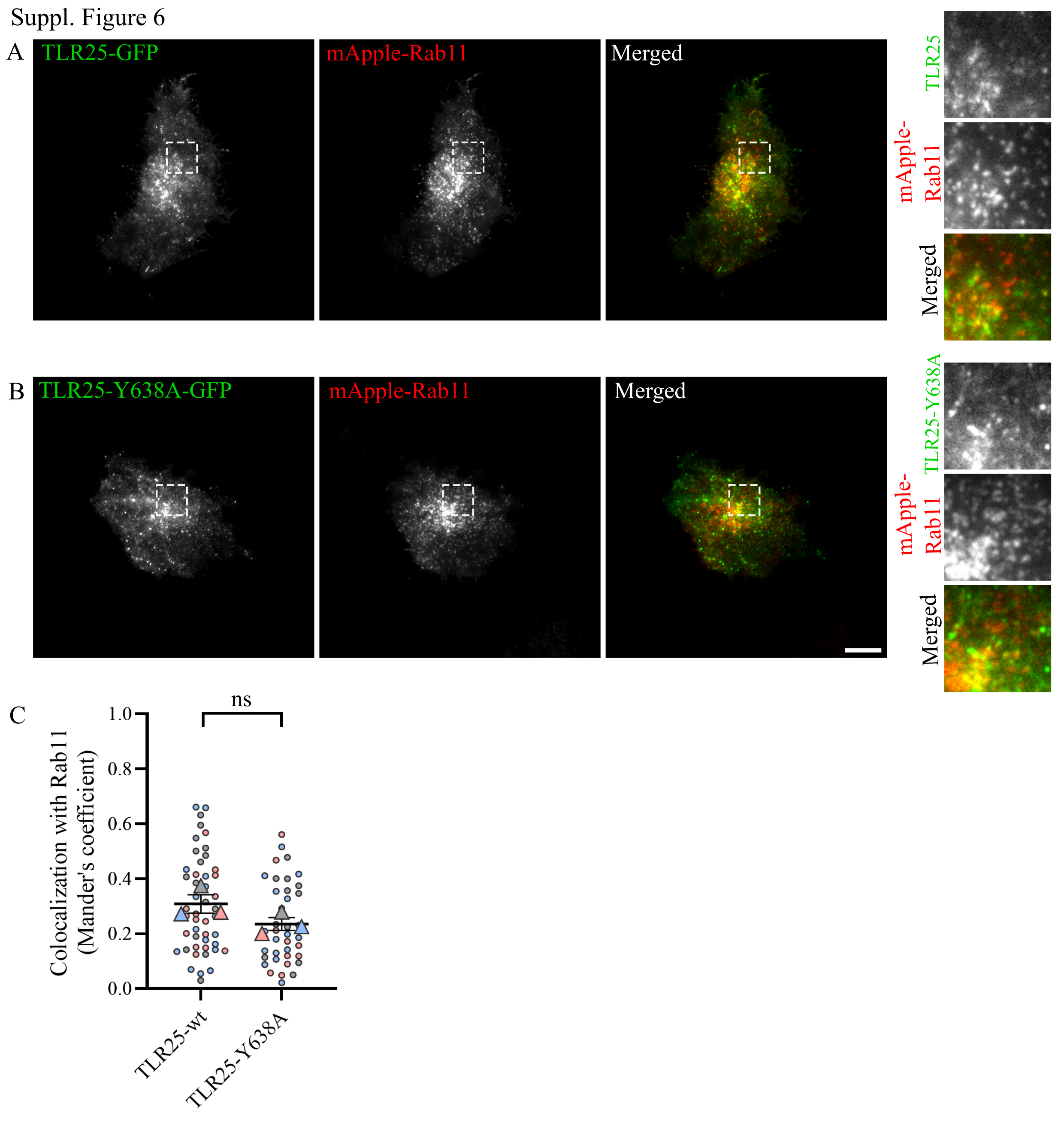

Supplement: Supplementary Figure 6 — TLR25 localizes to recycling endosomes independently of the predicted cytosolic sorting signal. Representative images of ACL cells transiently co-transfected with mApple-Rab11 and (A) TLR25-GFP wild-type or (B) TLR25-Y638A-GFP and imaged using a Leica Total Internal Reflection Fluorescence (TIRF) microscope with an optical section of 100 nm. Scale bar: 10 µm. Magnification of boxed areas are shown to the right. (C) The graph represents colocalization (Manders’ coefficient) between TLR25 wild-type or TLR25-Y638A and mApple-Rab11. Scatter plot shows the mean ± SEM from three independent experiments, color coded for each experimental repeat. n ≥ 40 cells in total. [file Image6.tiff]

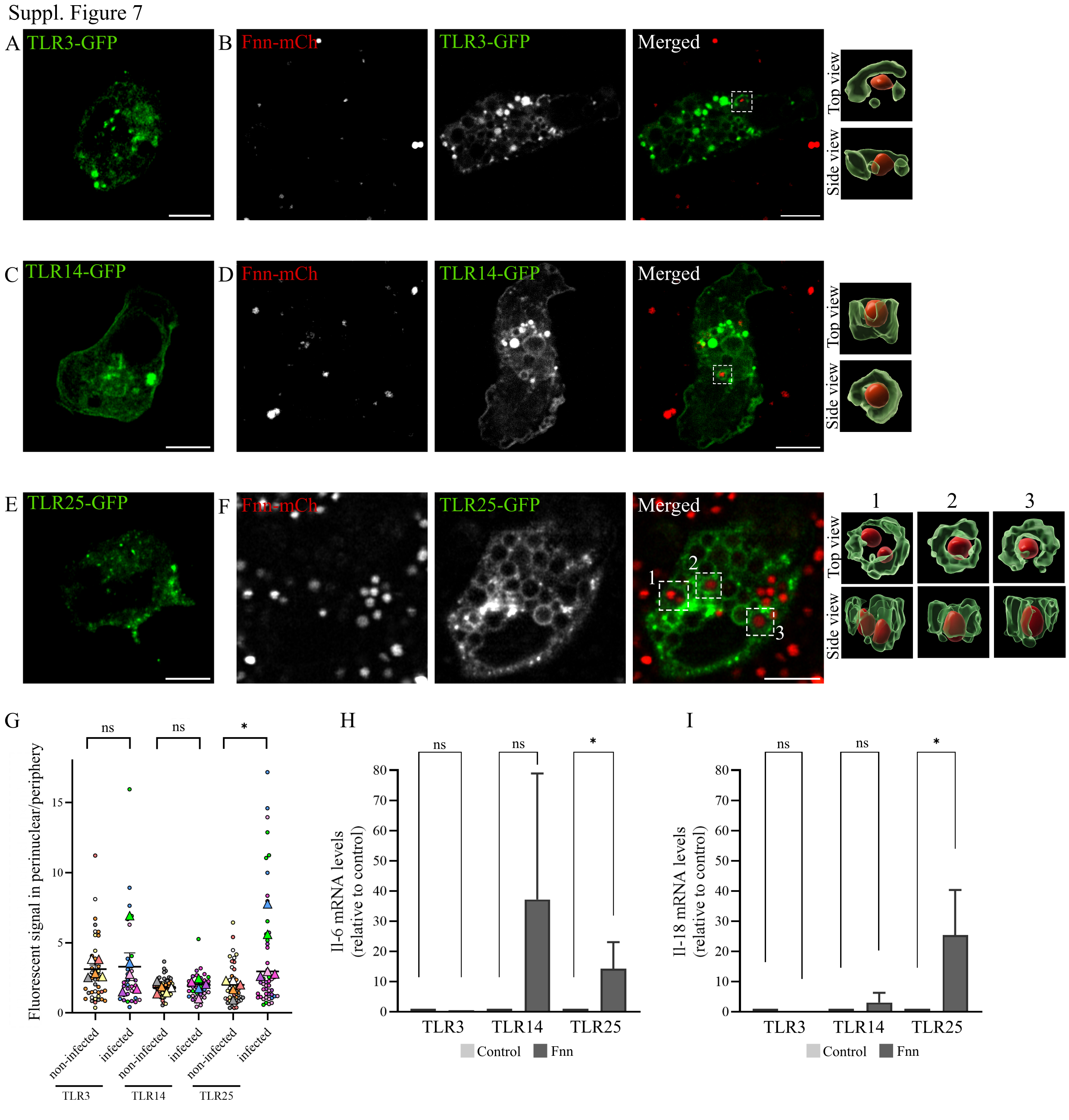

Supplement: Supplementary Figure 7 — Atlantic cod TLR25 responds to Francisella noatunensis subsp. noatunensis (Fnn) infection in primary leukocytes. Representative images of Atlantic cod primary leukocytes imaged using a Zeiss LSM880 Fast AiryScan microscope. Scale bars: 5 µm. The cells were transfected with TLR3-GFP and either non-infected (A) or infected with mCherry-expressing Fnn (B), transfected with TLR14-GFP and non-infected (C) or infected with mCherry-Fnn (D) or transfected with TLR25-GFP and non-infected (E) or infected with mCherry-Fnn (F). Magnification of boxed areas are shown to the right as IMARIS isosurface 3D rendering of a TLR-positive vesicle containing Fnn-mCherry. (G) The graph represents the ratio between the total fluorescence intensity from TLR3-GFP, TLR14-GFP, or TLR25-GFP in the perinuclear region over the peripheral region in non-infected vs. infected cells. Scatter plot shows the mean ± SEM from 5 fish per condition. Dots represent individual measurements, and the colors represent individual fish. n ≥ 52 cells in total. Quantification of mRNA levels of IL-6 (H) and IL-18 (I) in control or Fnn infected primary leukocytes expressing either TLR3-GFP, TLR14-GFP, or TLR25-GFP. Levels of mRNA were normalized to the amount of 18s and to the levels of cytokines in infected leukocytes expressing only GFP. Data represents the mean ± SD from three fish (TLR14 and TLR25) and one fish (TLR3). ns > 0.05, *p < 0.05, **p < 0.01 (two-tailed unpaired Student t-test). [file Image7.tiff]

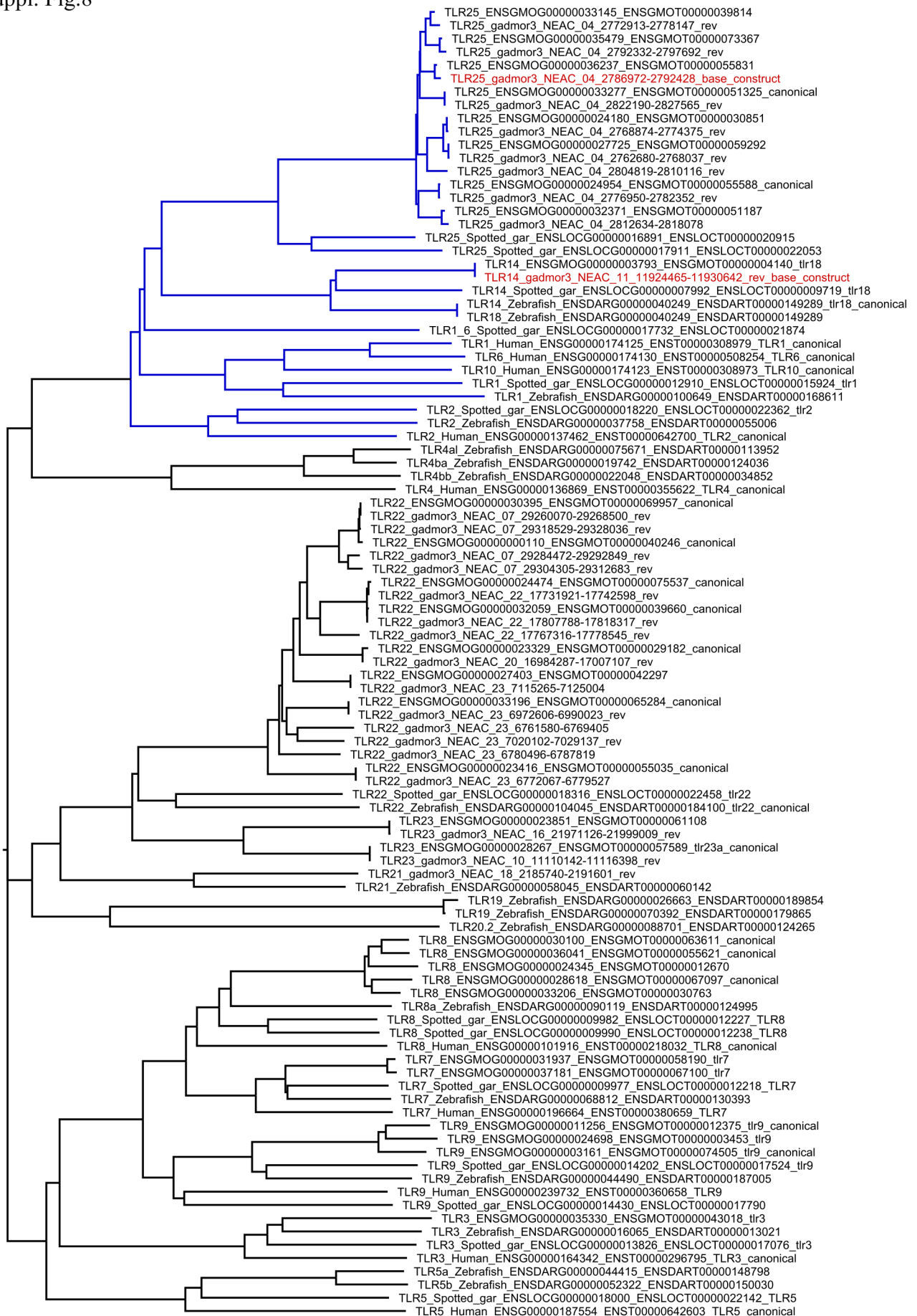

Supplement: Supplementary Figure 8 — Phylogenetic tree displayed as cladogram for TLR3, TLR14, TLR22 and TLR25 sequences manually annotated in the reference genome gadmor3 with available gene models from Ensembl.org. Sequences forming the base of constructs highlighted in blue. The tree was inferred using the Neighbor-Joining method, poisson distribution and pairwise deletion on a protein sequence alignment (ClustalW) using MEGAX. The most similar gene models for each construct are TLR3 ENSGMOG00000035330, TLR14 ENSGMOG00000003793 and TLR25 ENSGMOG00000036237. [file Image8.pdf]

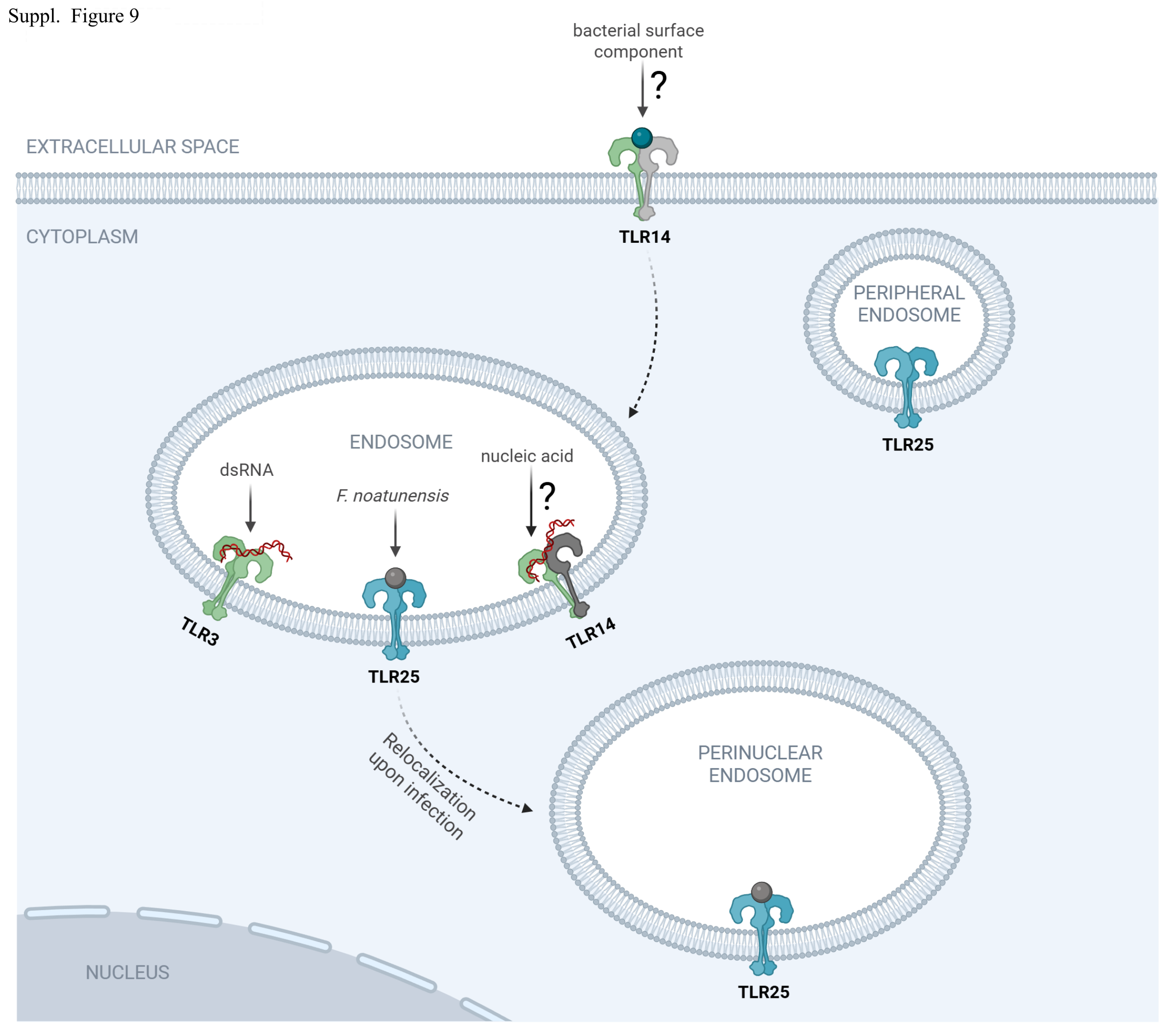

Supplement: Supplementary Figure 9 — Schematic overview of proposed TLR trafficking. TLR3 localizes to endosomes and is predicted to recognize dsRNA, similar to its mammalian ortholog. TLR14 localizes to both endosomes and the plasma membrane and may recognize nucleic acids and bacterial surface components in these compartments, respectively. TLR25 localizes to endosomes and small peripheral endosomes and responds to Fnn infection by relocalizing to the perinuclear region of the cell. Created in BioRender.com. Krokene, P. (2026). [file Image9.tiff]
